# Supplementary material for: Effects of antidiabetic agents on Alzheimer’s disease biomarkers in experimentally induced hyperglycemic rat model by streptozocin
Source: PLoS One. 2022 Jul 8;17(7):e0271138. doi: 10.1371/journal.pone.0271138 (PMC9269384; doi:10.1371/journal.pone.0271138)
Supplement: S1 Data — (DOC) [file pone.0271138.s001.doc]

Oneway

	

Descriptives	
	N	Mean	Std. Deviation	Std. Error	95% Confidence Interval for Mean	Minimum	Maximum	
					Lower Bound	Upper Bound			
Serum Glucose	Negative Control	6	132.1667	4.07022	1.66166	127.8952	136.4381	126.00	137.00	
	Positive Control	6	761.0000	92.97742	37.95787	663.4262	858.5738	649.00	882.00	
	Metformin	6	326.8333	41.80630	17.06735	282.9603	370.7064	268.00	379.00	
	Donepezil	6	676.3333	22.09676	9.02096	653.1442	699.5225	640.00	696.00	
	Insulin Glargin	6	695.6667	55.70159	22.74008	637.2114	754.1219	620.00	790.00	
	Glibenclamide	6	450.1667	40.69111	16.61208	407.4640	492.8694	400.00	493.00	
	Total	36	507.0278	233.49084	38.91514	428.0258	586.0297	126.00	882.00	
Serum HBA1c	Negative Control	6	5.40	.155	.063	5.24	5.56	5	6	
	Positive Control	6	9.68	2.086	.851	7.49	11.87	6	12	
	Metformin	6	8.57	.952	.389	7.57	9.57	8	10	
	Donepezil	6	10.13	.924	.377	9.16	11.10	9	11	
	Insulin Glargin	6	9.40	1.403	.573	7.93	10.87	8	11	
	Glibenclamide	6	8.53	.918	.375	7.57	9.50	8	10	
	Total	36	8.62	1.936	.323	7.96	9.27	5	12	
Serum Total Cholesterol	Negative Control	6	41.1667	6.76511	2.76184	34.0671	48.2662	33.00	50.00	
	Positive Control	6	76.5000	2.58844	1.05672	73.7836	79.2164	73.00	80.00	
	Metformin	6	60.0000	2.82843	1.15470	57.0317	62.9683	55.00	63.00	
	Donepezil	6	56.8333	14.35851	5.86184	41.7650	71.9017	41.00	75.00	
	Insulin Glargin	6	61.1667	6.46271	2.63839	54.3845	67.9489	50.00	68.00	
	Glibenclamide	6	41.1667	2.92689	1.19490	38.0951	44.2382	38.00	46.00	
	Total	36	56.1389	14.14850	2.35808	51.3517	60.9261	33.00	80.00	
Serum Triglyceride	Negative Control	6	60.1667	8.37655	3.41971	51.3760	68.9573	52.00	75.00	
	Positive Control	6	128.3333	65.39929	26.69915	59.7010	196.9657	69.00	254.00	
	Metformin	6	61.0000	25.69047	10.48809	34.0395	87.9605	36.00	107.00	
	Donepezil	6	65.3333	3.38625	1.38243	61.7797	68.8870	60.00	69.00	
	Insulin Glargin	6	68.1667	7.83369	3.19809	59.9457	76.3876	61.00	83.00	
	Glibenclamide	6	74.1667	4.62241	1.88709	69.3157	79.0176	69.00	80.00	
	Total	36	76.1944	36.19931	6.03322	63.9464	88.4425	36.00	254.00	
Serum Low Density Lipid	Negative Control	6	10.7667	7.58463	3.09641	2.8071	18.7263	4.00	24.00	
	Positive Control	6	14.3667	5.30723	2.16667	8.7971	19.9363	9.00	23.00	
	Metformin	6	13.3333	6.70840	2.73869	6.2933	20.3734	5.00	22.00	
	Donepezil	6	11.6333	10.46244	4.27127	.6537	22.6130	2.00	29.40	
	Insulin Glargin	6	13.5333	7.64373	3.12054	5.5117	21.5549	5.00	27.00	
	Glibenclamide	6	5.7333	5.63087	2.29879	-.1759	11.6426	2.00	17.00	
	Total	36	11.5611	7.46126	1.24354	9.0366	14.0856	2.00	29.40	
Serum High Density Lipid	Negative Control	6	29.5000	1.87083	.76376	27.5367	31.4633	27.00	32.00	
	Positive Control	6	34.1667	5.98052	2.44154	27.8905	40.4428	23.00	39.00	
	Metformin	6	34.8333	1.32916	.54263	33.4385	36.2282	33.00	37.00	
	Donepezil	6	34.1667	6.73548	2.74975	27.0982	41.2351	25.00	44.00	
	Insulin Glargin	6	36.0000	4.51664	1.84391	31.2601	40.7399	28.00	40.00	
	Glibenclamide	6	24.5000	2.73861	1.11803	21.6260	27.3740	21.00	29.00	
	Total	36	32.1944	5.72124	.95354	30.2587	34.1302	21.00	44.00	
Serum Interlukin 6	Negative Control	6	32.2833	8.12291	3.31616	23.7589	40.8078	18.70	40.00	
	Positive Control	6	81.6000	1.85580	.75763	79.6525	83.5475	79.10	84.00	
	Metformin	6	92.1000	3.75553	1.53319	88.1588	96.0412	86.30	96.00	
	Donepezil	6	106.9167	29.38628	11.99690	76.0777	137.7557	87.20	165.90	
	Insulin Glargin	6	117.4000	21.72556	8.86942	94.6004	140.1996	96.00	153.00	
	Glibenclamide	6	95.9833	5.83727	2.38305	89.8575	102.1092	85.70	101.40	
	Total	36	87.7139	31.14535	5.19089	77.1758	98.2520	18.70	165.90	
Serum Tumor Necrosis Factor	Negative Control	6	11.9333	1.50422	.61409	10.3548	13.5119	10.30	14.00	
	Positive Control	6	44.7167	2.89027	1.17995	41.6835	47.7498	40.20	48.00	
	Metformin	6	22.2667	4.09471	1.67166	17.9695	26.5638	19.00	30.00	
	Donepezil	6	21.2000	4.88016	1.99232	16.0786	26.3214	18.40	31.10	
	Insulin Glargin	6	63.0667	24.91439	10.17126	36.9206	89.2127	15.50	90.00	
	Glibenclamide	6	28.3833	6.04431	2.46758	22.0402	34.7264	22.70	39.10	
	Total	36	31.9278	20.04708	3.34118	25.1448	38.7107	10.30	90.00	
Serom Amyloid Beta 1 42	Negative Control	6	71.9167	2.08463	.85105	69.7290	74.1043	70.00	75.40	
	Positive Control	6	700.1000	49.10642	20.04761	648.5660	751.6340	610.10	741.50	
	Metformin	6	477.1667	18.32394	7.48072	457.9369	496.3965	450.00	493.00	
	Donepezil	6	546.1667	14.27469	5.82762	531.1863	561.1470	523.00	560.00	
	Insulin Glargin	6	577.6667	19.86622	8.11035	556.8183	598.5150	553.00	603.00	
	Glibenclamide	6	676.0000	30.44339	12.42846	644.0516	707.9484	636.00	708.00	
	Total	36	508.1694	213.58962	35.59827	435.9011	580.4378	70.00	741.50	
Serum Total Plasma Tau	Negative Control	6	22.9333	1.26754	.51747	21.6031	24.2635	21.00	24.60	
	Positive Control	6	140.8333	19.71209	8.04743	120.1468	161.5199	121.00	170.00	
	Metformin	6	90.1667	3.60093	1.47007	86.3877	93.9456	85.00	95.00	
	Donepezil	6	115.0667	4.10203	1.67465	110.7618	119.3715	110.00	120.00	
	Insulin Glargin	6	93.9000	5.04183	2.05832	88.6089	99.1911	88.00	100.00	
	Glibenclamide	6	106.9000	17.57214	7.17379	88.4592	125.3408	88.40	139.00	
	Total	36	94.9667	38.15371	6.35895	82.0573	107.8760	21.00	170.00	
Serum Neurofilament Light	Negative Control	6	3.4200	.38367	.15663	3.0174	3.8226	3.10	4.00	
	Positive Control	6	18.4167	.35449	.14472	18.0446	18.7887	17.90	19.00	
	Metformin	6	9.8333	.94733	.38674	8.8392	10.8275	8.10	11.00	
	Donepezil	6	7.4333	2.74275	1.11972	4.5550	10.3117	3.20	11.30	
	Insulin Glargin	6	3.1950	.38801	.15840	2.7878	3.6022	2.80	3.90	
	Glibenclamide	6	4.3833	.68120	.27810	3.6685	5.0982	3.10	4.97	
	Total	36	7.7803	5.50533	.91755	5.9175	9.6430	2.80	19.00	
Brain Amyloid Beta 1 42	Negative Control	6	86.0000	4.81664	1.96638	80.9452	91.0548	80.00	92.00	
	Positive Control	6	778.0000	38.83813	15.85560	737.2419	818.7581	700.00	799.00	
	Metformin	6	443.1667	23.37021	9.54085	418.6411	467.6922	419.00	480.00	
	Donepezil	6	531.7667	24.03944	9.81406	506.5388	556.9945	510.00	570.00	
	Insulin Glargin	6	546.6667	21.52828	8.78888	524.0741	569.2592	520.00	575.00	
	Glibenclamide	6	703.7333	10.19542	4.16226	693.0339	714.4328	695.00	721.00	
	Total	36	514.8889	226.00633	37.66772	438.4193	591.3584	80.00	799.00	
Brain Nitric Oxide	Negative Control	6	7.4383	.47373	.19340	6.9412	7.9355	6.93	8.10	
	Positive Control	6	29.8650	.61168	.24972	29.2231	30.5069	28.70	30.40	
	Metformin	6	9.6267	.35109	.14333	9.2582	9.9951	9.20	10.00	
	Donepezil	6	8.9867	.45218	.18460	8.5121	9.4612	8.15	9.40	
	Insulin Glargin	6	26.6667	2.67407	1.09169	23.8604	29.4729	23.10	30.90	
	Glibenclamide	6	24.4000	2.91204	1.18884	21.3440	27.4560	21.00	28.40	
	Total	36	17.8306	9.56221	1.59370	14.5952	21.0659	6.93	30.90	
Brain Acetyl choline Esterase	Negative Control	6	41.4883	1.37303	.56054	40.0474	42.9292	40.10	43.70	
	Positive Control	6	84.9083	3.34820	1.36690	81.3946	88.4220	81.00	89.00	
	Metformin	6	46.2500	1.89077	.77190	44.2658	48.2342	44.00	49.00	
	Donepezil	6	53.8167	2.16372	.88333	51.5460	56.0873	50.90	57.00	
	Insulin Glargin	6	53.6333	2.28619	.93333	51.2341	56.0325	50.30	57.00	
	Glibenclamide	6	72.3000	1.25698	.51316	70.9809	73.6191	70.80	74.00	
	Total	36	58.7328	15.47011	2.57835	53.4984	63.9671	40.10	89.00	
Brain Malondialdehyde	Negative Control	6	.3583	.10284	.04199	.2504	.4663	.22	.50	
	Positive Control	6	4.3233	.40604	.16576	3.8972	4.7494	3.76	4.90	
	Metformin	6	.9750	.03391	.01384	.9394	1.0106	.91	1.00	
	Donepezil	6	3.1500	.58907	.24049	2.5318	3.7682	2.40	3.80	
	Insulin Glargin	6	2.6517	.32065	.13090	2.3152	2.9882	2.10	2.90	
	Glibenclamide	6	3.5583	.47161	.19253	3.0634	4.0533	3.10	4.20	
	Total	36	2.5028	1.46442	.24407	2.0073	2.9983	.22	4.90	
Brain Beta Secretase	Negative Control	6	116.3667	2.31229	.94399	113.9401	118.7933	113.00	119.00	
	Positive Control	6	862.0333	20.53306	8.38259	840.4852	883.5815	836.00	897.00	
	Metformin	6	245.5833	13.89394	5.67218	231.0025	260.1641	219.00	260.00	
	Donepezil	6	187.0500	4.75973	1.94315	182.0550	192.0450	178.00	191.00	
	Insulin Glargin	6	317.9883	2.61404	1.06718	315.2451	320.7316	312.90	320.40	
	Glibenclamide	6	745.5333	8.81967	3.60062	736.2777	754.7890	734.20	758.00	
	Total	36	412.4258	289.50610	48.25102	314.4711	510.3806	113.00	897.00	
Brain PMAPt	Negative Control	6	16.3800	1.67331	.68313	14.6240	18.1360	14.33	19.10	
	Positive Control	6	44.5500	2.19431	.89582	42.2472	46.8528	42.30	48.00	
	Metformin	6	24.2667	1.58451	.64687	22.6038	25.9295	22.00	26.30	
	Donepezil	6	34.2417	3.01304	1.23007	31.0797	37.4037	30.90	38.30	
	Insulin Glargin	6	18.5333	.93310	.38093	17.5541	19.5126	17.40	20.00	
	Glibenclamide	6	20.8167	2.81668	1.14990	17.8607	23.7726	16.70	25.00	
	Total	36	26.4647	10.24477	1.70746	22.9984	29.9311	14.33	48.00	
Day0BS	Negative Control	6	80.3333	3.38625	1.38243	76.7797	83.8870	75.00	84.00	
	Positive Control	6	79.0000	2.96648	1.21106	75.8869	82.1131	75.00	82.00	
	Metformin	6	80.6667	3.72380	1.52023	76.7588	84.5746	77.00	85.00	
	Donepezil	6	76.5000	1.37840	.56273	75.0535	77.9465	75.00	78.00	
	Insulin Glargin	6	81.5000	1.87083	.76376	79.5367	83.4633	79.00	84.00	
	Glibenclamide	6	78.3333	3.55903	1.45297	74.5984	82.0683	75.00	85.00	
	Total	36	79.3889	3.20961	.53493	78.3029	80.4749	75.00	85.00	
Day3BS	Negative Control	6	175.0000	18.70829	7.63763	155.3669	194.6331	150.00	200.00	
	Positive Control	6	179.0000	17.20465	7.02377	160.9448	197.0552	155.00	199.00	
	Metformin	6	179.6667	16.75311	6.83943	162.0854	197.2480	156.00	198.00	
	Donepezil	6	175.3333	17.68238	7.21880	156.7768	193.8899	151.00	197.00	
	Insulin Glargin	6	176.0000	17.20465	7.02377	157.9448	194.0552	152.00	196.00	
	Glibenclamide	6	182.5000	16.53784	6.75154	165.1446	199.8554	159.00	200.00	
	Total	36	177.9167	16.30841	2.71807	172.3987	183.4346	150.00	200.00	
Day10BS	Negative Control	6	217.5000	15.41104	6.29153	201.3271	233.6729	200.00	240.00	
	Positive Control	6	237.0000	13.31165	5.43446	223.0303	250.9697	218.00	250.00	
	Metformin	6	234.6667	16.10797	6.57605	217.7624	251.5709	214.00	250.00	
	Donepezil	6	233.5000	10.63485	4.34166	222.3394	244.6606	218.00	247.00	
	Insulin Glargin	6	245.3333	3.72380	1.52023	241.4254	249.2412	240.00	250.00	
	Glibenclamide	6	238.8333	3.06050	1.24944	235.6215	242.0451	235.00	244.00	
	Total	36	234.4722	13.78091	2.29682	229.8094	239.1350	200.00	250.00	
Day30BS	Negative Control	6	353.3333	36.28590	14.81366	315.2536	391.4131	300.00	400.00	
	Positive Control	6	354.0000	26.19160	10.69268	326.5136	381.4864	325.00	399.00	
	Metformin	6	366.0000	34.41511	14.04991	329.8836	402.1164	318.00	400.00	
	Donepezil	6	363.0000	18.70829	7.63763	343.3669	382.6331	338.00	388.00	
	Insulin Glargin	6	390.1667	15.61303	6.37399	373.7818	406.5515	359.00	400.00	
	Glibenclamide	6	376.0000	14.43607	5.89350	360.8503	391.1497	360.00	398.00	
	Total	36	367.0833	27.19703	4.53284	357.8812	376.2855	300.00	400.00	
Day60BS	Negative Control	6	503.3333	48.44241	19.77653	452.4961	554.1705	420.00	550.00	
	Positive Control	6	576.1667	33.69520	13.75601	540.8057	611.5276	510.00	600.00	
	Metformin	6	505.8333	35.97453	14.68654	468.0804	543.5863	455.00	555.00	
	Donepezil	6	539.8333	27.84541	11.36784	510.6114	569.0553	516.00	580.00	
	Insulin Glargin	6	561.1667	34.37101	14.03191	525.0965	597.2368	518.00	599.00	
	Glibenclamide	6	533.3333	37.80300	15.43301	493.6615	573.0051	469.00	569.00	
	Total	36	536.6111	43.51373	7.25229	521.8882	551.3340	420.00	600.00	


Test of Homogeneity of Variances	
	Levene Statistic	df1	df2	Sig.	
Serum Glucose	Based on Mean	5.638	5	30	.001	
	Based on Median	5.248	5	30	.001	
	Based on Median and with adjusted df	5.248	5	16.481	.005	
	Based on trimmed mean	5.619	5	30	.001	
Serum HBA1c	Based on Mean	2.116	5	30	.091	
	Based on Median	1.625	5	30	.184	
	Based on Median and with adjusted df	1.625	5	9.746	.242	
	Based on trimmed mean	2.084	5	30	.095	
Serum Total Cholesterol	Based on Mean	7.284	5	30	.000	
	Based on Median	3.050	5	30	.024	
	Based on Median and with adjusted df	3.050	5	9.017	.070	
	Based on trimmed mean	7.087	5	30	.000	
Serum Triglyceride	Based on Mean	3.470	5	30	.014	
	Based on Median	2.479	5	30	.054	
	Based on Median and with adjusted df	2.479	5	6.510	.141	
	Based on trimmed mean	3.064	5	30	.024	
Serum Low Density Lipid	Based on Mean	.966	5	30	.454	
	Based on Median	.414	5	30	.835	
	Based on Median and with adjusted df	.414	5	21.071	.834	
	Based on trimmed mean	.905	5	30	.491	
Serum High Density Lipid	Based on Mean	2.843	5	30	.032	
	Based on Median	1.796	5	30	.144	
	Based on Median and with adjusted df	1.796	5	15.890	.171	
	Based on trimmed mean	2.665	5	30	.041	
Serum Interlukin 6	Based on Mean	3.982	5	30	.007	
	Based on Median	1.884	5	30	.127	
	Based on Median and with adjusted df	1.884	5	7.444	.210	
	Based on trimmed mean	3.167	5	30	.021	
Serum Tumor Necrosis Factor	Based on Mean	3.003	5	30	.026	
	Based on Median	1.455	5	30	.234	
	Based on Median and with adjusted df	1.455	5	6.102	.326	
	Based on trimmed mean	2.467	5	30	.055	
Serom Amyloid Beta 1 42	Based on Mean	3.844	5	30	.008	
	Based on Median	2.863	5	30	.031	
	Based on Median and with adjusted df	2.863	5	11.150	.067	
	Based on trimmed mean	3.795	5	30	.009	
Serum Total Plasma Tau	Based on Mean	6.194	5	30	.000	
	Based on Median	3.272	5	30	.018	
	Based on Median and with adjusted df	3.272	5	10.305	.051	
	Based on trimmed mean	5.591	5	30	.001	
Serum Neurofilament Light	Based on Mean	4.642	5	30	.003	
	Based on Median	4.422	5	30	.004	
	Based on Median and with adjusted df	4.422	5	9.252	.025	
	Based on trimmed mean	4.599	5	30	.003	
Brain Amyloid Beta 1 42	Based on Mean	2.067	5	30	.097	
	Based on Median	.780	5	30	.572	
	Based on Median and with adjusted df	.780	5	10.211	.586	
	Based on trimmed mean	1.647	5	30	.178	
Brain Nitric Oxide	Based on Mean	7.479	5	30	.000	
	Based on Median	7.072	5	30	.000	
	Based on Median and with adjusted df	7.072	5	11.541	.003	
	Based on trimmed mean	7.474	5	30	.000	
Brain Acetyl choline Esterase	Based on Mean	2.825	5	30	.033	
	Based on Median	2.636	5	30	.043	
	Based on Median and with adjusted df	2.636	5	22.195	.051	
	Based on trimmed mean	2.831	5	30	.033	
Brain Malondialdehyde	Based on Mean	6.075	5	30	.001	
	Based on Median	3.063	5	30	.024	
	Based on Median and with adjusted df	3.063	5	18.520	.035	
	Based on trimmed mean	5.827	5	30	.001	
Brain Beta Secretase	Based on Mean	2.790	5	30	.035	
	Based on Median	2.350	5	30	.065	
	Based on Median and with adjusted df	2.350	5	12.669	.101	
	Based on trimmed mean	2.727	5	30	.038	
Brain PMAPt	Based on Mean	1.668	5	30	.173	
	Based on Median	1.395	5	30	.254	
	Based on Median and with adjusted df	1.395	5	20.957	.267	
	Based on trimmed mean	1.640	5	30	.180	
Day0BS	Based on Mean	1.831	5	30	.137	
	Based on Median	1.522	5	30	.213	
	Based on Median and with adjusted df	1.522	5	15.705	.239	
	Based on trimmed mean	1.759	5	30	.152	
Day3BS	Based on Mean	.027	5	30	1.000	
	Based on Median	.027	5	30	1.000	
	Based on Median and with adjusted df	.027	5	29.596	1.000	
	Based on trimmed mean	.027	5	30	1.000	
Day10BS	Based on Mean	6.252	5	30	.000	
	Based on Median	4.629	5	30	.003	
	Based on Median and with adjusted df	4.629	5	21.377	.005	
	Based on trimmed mean	6.203	5	30	.000	
Day30BS	Based on Mean	2.251	5	30	.075	
	Based on Median	1.347	5	30	.272	
	Based on Median and with adjusted df	1.347	5	19.972	.286	
	Based on trimmed mean	2.230	5	30	.077	
Day60BS	Based on Mean	.462	5	30	.801	
	Based on Median	.508	5	30	.768	
	Based on Median and with adjusted df	.508	5	22.974	.767	
	Based on trimmed mean	.512	5	30	.765	


ANOVA	
	Sum of Squares	df	Mean Square	F	Sig.	
Serum Glucose	Between Groups	1829849.806	5	365969.961	140.256	.000	
	Within Groups	78279.167	30	2609.306			
	Total	1908128.972	35				
Serum HBA1c	Between Groups	86.448	5	17.290	11.596	.000	
	Within Groups	44.728	30	1.491			
	Total	131.176	35				
Serum Total Cholesterol	Between Groups	5421.472	5	1084.294	20.525	.000	
	Within Groups	1584.833	30	52.828			
	Total	7006.306	35				
Serum Triglyceride	Between Groups	20356.472	5	4071.294	4.788	.002	
	Within Groups	25507.167	30	850.239			
	Total	45863.639	35				
Serum Low Density Lipid	Between Groups	297.006	5	59.401	1.079	.392	
	Within Groups	1651.460	30	55.049			
	Total	1948.466	35				
Serum High Density Lipid	Between Groups	574.139	5	114.828	6.028	.001	
	Within Groups	571.500	30	19.050			
	Total	1145.639	35				
Serum Interlukin 6	Between Groups	26685.358	5	5337.072	22.036	.000	
	Within Groups	7265.785	30	242.193			
	Total	33951.143	35				
Serum Tumor Necrosis Factor	Between Groups	10523.696	5	2104.739	17.825	.000	
	Within Groups	3542.297	30	118.077			
	Total	14065.992	35				
Serom Amyloid Beta 1 42	Between Groups	1575334.468	5	315066.894	442.015	.000	
	Within Groups	21383.928	30	712.798			
	Total	1596718.396	35				
Serum Total Plasma Tau	Between Groups	47178.867	5	9435.773	75.069	.000	
	Within Groups	3770.833	30	125.694			
	Total	50949.700	35				
Serum Neurofilament Light	Between Groups	1014.265	5	202.853	130.767	.000	
	Within Groups	46.538	30	1.551			
	Total	1060.803	35				
Brain Amyloid Beta 1 42	Between Groups	1771644.742	5	354328.948	659.610	.000	
	Within Groups	16115.373	30	537.179			
	Total	1787760.116	35				
Brain Nitric Oxide	Between Groups	3117.473	5	623.495	225.945	.000	
	Within Groups	82.785	30	2.759			
	Total	3200.257	35				
Brain Acetyl choline Esterase	Between Groups	8235.554	5	1647.111	350.960	.000	
	Within Groups	140.795	30	4.693			
	Total	8376.349	35				
Brain Malondialdehyde	Between Groups	70.815	5	14.163	100.112	.000	
	Within Groups	4.244	30	.141			
	Total	75.059	35				
Brain Beta Secretase	Between Groups	2929846.009	5	585969.202	4834.265	.000	
	Within Groups	3636.349	30	121.212			
	Total	2933482.359	35				
Brain PMAPt	Between Groups	3533.392	5	706.678	151.386	.000	
	Within Groups	140.042	30	4.668			
	Total	3673.434	35				
Day0BS	Between Groups	99.556	5	19.911	2.289	.071	
	Within Groups	261.000	30	8.700			
	Total	360.556	35				
Day3BS	Between Groups	264.583	5	52.917	.176	.970	
	Within Groups	9044.167	30	301.472			
	Total	9308.750	35				
Day10BS	Between Groups	2594.472	5	518.894	3.841	.008	
	Within Groups	4052.500	30	135.083			
	Total	6646.972	35				
Day30BS	Between Groups	5942.583	5	1188.517	1.788	.146	
	Within Groups	19946.167	30	664.872			
	Total	25888.750	35				
Day60BS	Between Groups	25460.556	5	5092.111	3.743	.009	
	Within Groups	40810.000	30	1360.333			
	Total	66270.556	35				


Post Hoc Tests


Multiple Comparisons	
Tukey HSD  	
Dependent Variable	(I) Group	(J) Group	Mean Difference (I-J)	Std. Error	Sig.	95% Confidence Interval	
						Lower Bound	Upper Bound	
Serum Glucose	Negative Control	Positive Control	-628.83333*	29.49184	.000	-718.5355	-539.1311	
		Metformin	-194.66667*	29.49184	.000	-284.3689	-104.9645	
		Donepezil	-544.16667*	29.49184	.000	-633.8689	-454.4645	
		Insulin Glargin	-563.50000*	29.49184	.000	-653.2022	-473.7978	
		Glibenclamide	-318.00000*	29.49184	.000	-407.7022	-228.2978	
	Positive Control	Negative Control	628.83333*	29.49184	.000	539.1311	718.5355	
		Metformin	434.16667*	29.49184	.000	344.4645	523.8689	
		Donepezil	84.66667	29.49184	.073	-5.0355	174.3689	
		Insulin Glargin	65.33333	29.49184	.261	-24.3689	155.0355	
		Glibenclamide	310.83333*	29.49184	.000	221.1311	400.5355	
	Metformin	Negative Control	194.66667*	29.49184	.000	104.9645	284.3689	
		Positive Control	-434.16667*	29.49184	.000	-523.8689	-344.4645	
		Donepezil	-349.50000*	29.49184	.000	-439.2022	-259.7978	
		Insulin Glargin	-368.83333*	29.49184	.000	-458.5355	-279.1311	
		Glibenclamide	-123.33333*	29.49184	.003	-213.0355	-33.6311	
	Donepezil	Negative Control	544.16667*	29.49184	.000	454.4645	633.8689	
		Positive Control	-84.66667	29.49184	.073	-174.3689	5.0355	
		Metformin	349.50000*	29.49184	.000	259.7978	439.2022	
		Insulin Glargin	-19.33333	29.49184	.985	-109.0355	70.3689	
		Glibenclamide	226.16667*	29.49184	.000	136.4645	315.8689	
	Insulin Glargin	Negative Control	563.50000*	29.49184	.000	473.7978	653.2022	
		Positive Control	-65.33333	29.49184	.261	-155.0355	24.3689	
		Metformin	368.83333*	29.49184	.000	279.1311	458.5355	
		Donepezil	19.33333	29.49184	.985	-70.3689	109.0355	
		Glibenclamide	245.50000*	29.49184	.000	155.7978	335.2022	
	Glibenclamide	Negative Control	318.00000*	29.49184	.000	228.2978	407.7022	
		Positive Control	-310.83333*	29.49184	.000	-400.5355	-221.1311	
		Metformin	123.33333*	29.49184	.003	33.6311	213.0355	
		Donepezil	-226.16667*	29.49184	.000	-315.8689	-136.4645	
		Insulin Glargin	-245.50000*	29.49184	.000	-335.2022	-155.7978	
Serum HBA1c	Negative Control	Positive Control	-4.283*	.705	.000	-6.43	-2.14	
		Metformin	-3.167*	.705	.001	-5.31	-1.02	
		Donepezil	-4.733*	.705	.000	-6.88	-2.59	
		Insulin Glargin	-4.000*	.705	.000	-6.14	-1.86	
		Glibenclamide	-3.133*	.705	.001	-5.28	-.99	
	Positive Control	Negative Control	4.283*	.705	.000	2.14	6.43	
		Metformin	1.117	.705	.615	-1.03	3.26	
		Donepezil	-.450	.705	.987	-2.59	1.69	
		Insulin Glargin	.283	.705	.999	-1.86	2.43	
		Glibenclamide	1.150	.705	.585	-.99	3.29	
	Metformin	Negative Control	3.167*	.705	.001	1.02	5.31	
		Positive Control	-1.117	.705	.615	-3.26	1.03	
		Donepezil	-1.567	.705	.258	-3.71	.58	
		Insulin Glargin	-.833	.705	.842	-2.98	1.31	
		Glibenclamide	.033	.705	1.000	-2.11	2.18	
	Donepezil	Negative Control	4.733*	.705	.000	2.59	6.88	
		Positive Control	.450	.705	.987	-1.69	2.59	
		Metformin	1.567	.705	.258	-.58	3.71	
		Insulin Glargin	.733	.705	.900	-1.41	2.88	
		Glibenclamide	1.600	.705	.238	-.54	3.74	
	Insulin Glargin	Negative Control	4.000*	.705	.000	1.86	6.14	
		Positive Control	-.283	.705	.999	-2.43	1.86	
		Metformin	.833	.705	.842	-1.31	2.98	
		Donepezil	-.733	.705	.900	-2.88	1.41	
		Glibenclamide	.867	.705	.819	-1.28	3.01	
	Glibenclamide	Negative Control	3.133*	.705	.001	.99	5.28	
		Positive Control	-1.150	.705	.585	-3.29	.99	
		Metformin	-.033	.705	1.000	-2.18	2.11	
		Donepezil	-1.600	.705	.238	-3.74	.54	
		Insulin Glargin	-.867	.705	.819	-3.01	1.28	
Serum Total Cholesterol	Negative Control	Positive Control	-35.33333*	4.19634	.000	-48.0969	-22.5698	
		Metformin	-18.83333*	4.19634	.001	-31.5969	-6.0698	
		Donepezil	-15.66667*	4.19634	.009	-28.4302	-2.9031	
		Insulin Glargin	-20.00000*	4.19634	.001	-32.7636	-7.2364	
		Glibenclamide	.00000	4.19634	1.000	-12.7636	12.7636	
	Positive Control	Negative Control	35.33333*	4.19634	.000	22.5698	48.0969	
		Metformin	16.50000*	4.19634	.006	3.7364	29.2636	
		Donepezil	19.66667*	4.19634	.001	6.9031	32.4302	
		Insulin Glargin	15.33333*	4.19634	.011	2.5698	28.0969	
		Glibenclamide	35.33333*	4.19634	.000	22.5698	48.0969	
	Metformin	Negative Control	18.83333*	4.19634	.001	6.0698	31.5969	
		Positive Control	-16.50000*	4.19634	.006	-29.2636	-3.7364	
		Donepezil	3.16667	4.19634	.973	-9.5969	15.9302	
		Insulin Glargin	-1.16667	4.19634	1.000	-13.9302	11.5969	
		Glibenclamide	18.83333*	4.19634	.001	6.0698	31.5969	
	Donepezil	Negative Control	15.66667*	4.19634	.009	2.9031	28.4302	
		Positive Control	-19.66667*	4.19634	.001	-32.4302	-6.9031	
		Metformin	-3.16667	4.19634	.973	-15.9302	9.5969	
		Insulin Glargin	-4.33333	4.19634	.903	-17.0969	8.4302	
		Glibenclamide	15.66667*	4.19634	.009	2.9031	28.4302	
	Insulin Glargin	Negative Control	20.00000*	4.19634	.001	7.2364	32.7636	
		Positive Control	-15.33333*	4.19634	.011	-28.0969	-2.5698	
		Metformin	1.16667	4.19634	1.000	-11.5969	13.9302	
		Donepezil	4.33333	4.19634	.903	-8.4302	17.0969	
		Glibenclamide	20.00000*	4.19634	.001	7.2364	32.7636	
	Glibenclamide	Negative Control	.00000	4.19634	1.000	-12.7636	12.7636	
		Positive Control	-35.33333*	4.19634	.000	-48.0969	-22.5698	
		Metformin	-18.83333*	4.19634	.001	-31.5969	-6.0698	
		Donepezil	-15.66667*	4.19634	.009	-28.4302	-2.9031	
		Insulin Glargin	-20.00000*	4.19634	.001	-32.7636	-7.2364	
Serum Triglyceride	Negative Control	Positive Control	-68.16667*	16.83487	.004	-119.3715	-16.9618	
		Metformin	-.83333	16.83487	1.000	-52.0382	50.3715	
		Donepezil	-5.16667	16.83487	1.000	-56.3715	46.0382	
		Insulin Glargin	-8.00000	16.83487	.997	-59.2049	43.2049	
		Glibenclamide	-14.00000	16.83487	.959	-65.2049	37.2049	
	Positive Control	Negative Control	68.16667*	16.83487	.004	16.9618	119.3715	
		Metformin	67.33333*	16.83487	.005	16.1285	118.5382	
		Donepezil	63.00000*	16.83487	.009	11.7951	114.2049	
		Insulin Glargin	60.16667*	16.83487	.014	8.9618	111.3715	
		Glibenclamide	54.16667*	16.83487	.033	2.9618	105.3715	
	Metformin	Negative Control	.83333	16.83487	1.000	-50.3715	52.0382	
		Positive Control	-67.33333*	16.83487	.005	-118.5382	-16.1285	
		Donepezil	-4.33333	16.83487	1.000	-55.5382	46.8715	
		Insulin Glargin	-7.16667	16.83487	.998	-58.3715	44.0382	
		Glibenclamide	-13.16667	16.83487	.968	-64.3715	38.0382	
	Donepezil	Negative Control	5.16667	16.83487	1.000	-46.0382	56.3715	
		Positive Control	-63.00000*	16.83487	.009	-114.2049	-11.7951	
		Metformin	4.33333	16.83487	1.000	-46.8715	55.5382	
		Insulin Glargin	-2.83333	16.83487	1.000	-54.0382	48.3715	
		Glibenclamide	-8.83333	16.83487	.995	-60.0382	42.3715	
	Insulin Glargin	Negative Control	8.00000	16.83487	.997	-43.2049	59.2049	
		Positive Control	-60.16667*	16.83487	.014	-111.3715	-8.9618	
		Metformin	7.16667	16.83487	.998	-44.0382	58.3715	
		Donepezil	2.83333	16.83487	1.000	-48.3715	54.0382	
		Glibenclamide	-6.00000	16.83487	.999	-57.2049	45.2049	
	Glibenclamide	Negative Control	14.00000	16.83487	.959	-37.2049	65.2049	
		Positive Control	-54.16667*	16.83487	.033	-105.3715	-2.9618	
		Metformin	13.16667	16.83487	.968	-38.0382	64.3715	
		Donepezil	8.83333	16.83487	.995	-42.3715	60.0382	
		Insulin Glargin	6.00000	16.83487	.999	-45.2049	57.2049	
Serum Low Density Lipid	Negative Control	Positive Control	-3.60000	4.28364	.957	-16.6291	9.4291	
		Metformin	-2.56667	4.28364	.990	-15.5958	10.4624	
		Donepezil	-.86667	4.28364	1.000	-13.8958	12.1624	
		Insulin Glargin	-2.76667	4.28364	.986	-15.7958	10.2624	
		Glibenclamide	5.03333	4.28364	.845	-7.9958	18.0624	
	Positive Control	Negative Control	3.60000	4.28364	.957	-9.4291	16.6291	
		Metformin	1.03333	4.28364	1.000	-11.9958	14.0624	
		Donepezil	2.73333	4.28364	.987	-10.2958	15.7624	
		Insulin Glargin	.83333	4.28364	1.000	-12.1958	13.8624	
		Glibenclamide	8.63333	4.28364	.358	-4.3958	21.6624	
	Metformin	Negative Control	2.56667	4.28364	.990	-10.4624	15.5958	
		Positive Control	-1.03333	4.28364	1.000	-14.0624	11.9958	
		Donepezil	1.70000	4.28364	.999	-11.3291	14.7291	
		Insulin Glargin	-.20000	4.28364	1.000	-13.2291	12.8291	
		Glibenclamide	7.60000	4.28364	.497	-5.4291	20.6291	
	Donepezil	Negative Control	.86667	4.28364	1.000	-12.1624	13.8958	
		Positive Control	-2.73333	4.28364	.987	-15.7624	10.2958	
		Metformin	-1.70000	4.28364	.999	-14.7291	11.3291	
		Insulin Glargin	-1.90000	4.28364	.998	-14.9291	11.1291	
		Glibenclamide	5.90000	4.28364	.740	-7.1291	18.9291	
	Insulin Glargin	Negative Control	2.76667	4.28364	.986	-10.2624	15.7958	
		Positive Control	-.83333	4.28364	1.000	-13.8624	12.1958	
		Metformin	.20000	4.28364	1.000	-12.8291	13.2291	
		Donepezil	1.90000	4.28364	.998	-11.1291	14.9291	
		Glibenclamide	7.80000	4.28364	.468	-5.2291	20.8291	
	Glibenclamide	Negative Control	-5.03333	4.28364	.845	-18.0624	7.9958	
		Positive Control	-8.63333	4.28364	.358	-21.6624	4.3958	
		Metformin	-7.60000	4.28364	.497	-20.6291	5.4291	
		Donepezil	-5.90000	4.28364	.740	-18.9291	7.1291	
		Insulin Glargin	-7.80000	4.28364	.468	-20.8291	5.2291	
Serum High Density Lipid	Negative Control	Positive Control	-4.66667	2.51992	.450	-12.3312	2.9979	
		Metformin	-5.33333	2.51992	.306	-12.9979	2.3312	
		Donepezil	-4.66667	2.51992	.450	-12.3312	2.9979	
		Insulin Glargin	-6.50000	2.51992	.134	-14.1646	1.1646	
		Glibenclamide	5.00000	2.51992	.374	-2.6646	12.6646	
	Positive Control	Negative Control	4.66667	2.51992	.450	-2.9979	12.3312	
		Metformin	-.66667	2.51992	1.000	-8.3312	6.9979	
		Donepezil	.00000	2.51992	1.000	-7.6646	7.6646	
		Insulin Glargin	-1.83333	2.51992	.977	-9.4979	5.8312	
		Glibenclamide	9.66667*	2.51992	.007	2.0021	17.3312	
	Metformin	Negative Control	5.33333	2.51992	.306	-2.3312	12.9979	
		Positive Control	.66667	2.51992	1.000	-6.9979	8.3312	
		Donepezil	.66667	2.51992	1.000	-6.9979	8.3312	
		Insulin Glargin	-1.16667	2.51992	.997	-8.8312	6.4979	
		Glibenclamide	10.33333*	2.51992	.004	2.6688	17.9979	
	Donepezil	Negative Control	4.66667	2.51992	.450	-2.9979	12.3312	
		Positive Control	.00000	2.51992	1.000	-7.6646	7.6646	
		Metformin	-.66667	2.51992	1.000	-8.3312	6.9979	
		Insulin Glargin	-1.83333	2.51992	.977	-9.4979	5.8312	
		Glibenclamide	9.66667*	2.51992	.007	2.0021	17.3312	
	Insulin Glargin	Negative Control	6.50000	2.51992	.134	-1.1646	14.1646	
		Positive Control	1.83333	2.51992	.977	-5.8312	9.4979	
		Metformin	1.16667	2.51992	.997	-6.4979	8.8312	
		Donepezil	1.83333	2.51992	.977	-5.8312	9.4979	
		Glibenclamide	11.50000*	2.51992	.001	3.8354	19.1646	
	Glibenclamide	Negative Control	-5.00000	2.51992	.374	-12.6646	2.6646	
		Positive Control	-9.66667*	2.51992	.007	-17.3312	-2.0021	
		Metformin	-10.33333*	2.51992	.004	-17.9979	-2.6688	
		Donepezil	-9.66667*	2.51992	.007	-17.3312	-2.0021	
		Insulin Glargin	-11.50000*	2.51992	.001	-19.1646	-3.8354	
Serum Interlukin 6	Negative Control	Positive Control	-49.31667*	8.98504	.000	-76.6455	-21.9878	
		Metformin	-59.81667*	8.98504	.000	-87.1455	-32.4878	
		Donepezil	-74.63333*	8.98504	.000	-101.9622	-47.3045	
		Insulin Glargin	-85.11667*	8.98504	.000	-112.4455	-57.7878	
		Glibenclamide	-63.70000*	8.98504	.000	-91.0288	-36.3712	
	Positive Control	Negative Control	49.31667*	8.98504	.000	21.9878	76.6455	
		Metformin	-10.50000	8.98504	.848	-37.8288	16.8288	
		Donepezil	-25.31667	8.98504	.082	-52.6455	2.0122	
		Insulin Glargin	-35.80000*	8.98504	.005	-63.1288	-8.4712	
		Glibenclamide	-14.38333	8.98504	.604	-41.7122	12.9455	
	Metformin	Negative Control	59.81667*	8.98504	.000	32.4878	87.1455	
		Positive Control	10.50000	8.98504	.848	-16.8288	37.8288	
		Donepezil	-14.81667	8.98504	.574	-42.1455	12.5122	
		Insulin Glargin	-25.30000	8.98504	.082	-52.6288	2.0288	
		Glibenclamide	-3.88333	8.98504	.998	-31.2122	23.4455	
	Donepezil	Negative Control	74.63333*	8.98504	.000	47.3045	101.9622	
		Positive Control	25.31667	8.98504	.082	-2.0122	52.6455	
		Metformin	14.81667	8.98504	.574	-12.5122	42.1455	
		Insulin Glargin	-10.48333	8.98504	.849	-37.8122	16.8455	
		Glibenclamide	10.93333	8.98504	.825	-16.3955	38.2622	
	Insulin Glargin	Negative Control	85.11667*	8.98504	.000	57.7878	112.4455	
		Positive Control	35.80000*	8.98504	.005	8.4712	63.1288	
		Metformin	25.30000	8.98504	.082	-2.0288	52.6288	
		Donepezil	10.48333	8.98504	.849	-16.8455	37.8122	
		Glibenclamide	21.41667	8.98504	.194	-5.9122	48.7455	
	Glibenclamide	Negative Control	63.70000*	8.98504	.000	36.3712	91.0288	
		Positive Control	14.38333	8.98504	.604	-12.9455	41.7122	
		Metformin	3.88333	8.98504	.998	-23.4455	31.2122	
		Donepezil	-10.93333	8.98504	.825	-38.2622	16.3955	
		Insulin Glargin	-21.41667	8.98504	.194	-48.7455	5.9122	
Serum Tumor Necrosis Factor	Negative Control	Positive Control	-32.78333*	6.27366	.000	-51.8653	-13.7014	
		Metformin	-10.33333	6.27366	.575	-29.4153	8.7486	
		Donepezil	-9.26667	6.27366	.681	-28.3486	9.8153	
		Insulin Glargin	-51.13333*	6.27366	.000	-70.2153	-32.0514	
		Glibenclamide	-16.45000	6.27366	.123	-35.5319	2.6319	
	Positive Control	Negative Control	32.78333*	6.27366	.000	13.7014	51.8653	
		Metformin	22.45000*	6.27366	.014	3.3681	41.5319	
		Donepezil	23.51667*	6.27366	.009	4.4347	42.5986	
		Insulin Glargin	-18.35000	6.27366	.065	-37.4319	.7319	
		Glibenclamide	16.33333	6.27366	.128	-2.7486	35.4153	
	Metformin	Negative Control	10.33333	6.27366	.575	-8.7486	29.4153	
		Positive Control	-22.45000*	6.27366	.014	-41.5319	-3.3681	
		Donepezil	1.06667	6.27366	1.000	-18.0153	20.1486	
		Insulin Glargin	-40.80000*	6.27366	.000	-59.8819	-21.7181	
		Glibenclamide	-6.11667	6.27366	.922	-25.1986	12.9653	
	Donepezil	Negative Control	9.26667	6.27366	.681	-9.8153	28.3486	
		Positive Control	-23.51667*	6.27366	.009	-42.5986	-4.4347	
		Metformin	-1.06667	6.27366	1.000	-20.1486	18.0153	
		Insulin Glargin	-41.86667*	6.27366	.000	-60.9486	-22.7847	
		Glibenclamide	-7.18333	6.27366	.858	-26.2653	11.8986	
	Insulin Glargin	Negative Control	51.13333*	6.27366	.000	32.0514	70.2153	
		Positive Control	18.35000	6.27366	.065	-.7319	37.4319	
		Metformin	40.80000*	6.27366	.000	21.7181	59.8819	
		Donepezil	41.86667*	6.27366	.000	22.7847	60.9486	
		Glibenclamide	34.68333*	6.27366	.000	15.6014	53.7653	
	Glibenclamide	Negative Control	16.45000	6.27366	.123	-2.6319	35.5319	
		Positive Control	-16.33333	6.27366	.128	-35.4153	2.7486	
		Metformin	6.11667	6.27366	.922	-12.9653	25.1986	
		Donepezil	7.18333	6.27366	.858	-11.8986	26.2653	
		Insulin Glargin	-34.68333*	6.27366	.000	-53.7653	-15.6014	
Serom Amyloid Beta 1 42	Negative Control	Positive Control	-628.18333*	15.41425	.000	-675.0672	-581.2994	
		Metformin	-405.25000*	15.41425	.000	-452.1339	-358.3661	
		Donepezil	-474.25000*	15.41425	.000	-521.1339	-427.3661	
		Insulin Glargin	-505.75000*	15.41425	.000	-552.6339	-458.8661	
		Glibenclamide	-604.08333*	15.41425	.000	-650.9672	-557.1994	
	Positive Control	Negative Control	628.18333*	15.41425	.000	581.2994	675.0672	
		Metformin	222.93333*	15.41425	.000	176.0494	269.8172	
		Donepezil	153.93333*	15.41425	.000	107.0494	200.8172	
		Insulin Glargin	122.43333*	15.41425	.000	75.5494	169.3172	
		Glibenclamide	24.10000	15.41425	.628	-22.7839	70.9839	
	Metformin	Negative Control	405.25000*	15.41425	.000	358.3661	452.1339	
		Positive Control	-222.93333*	15.41425	.000	-269.8172	-176.0494	
		Donepezil	-69.00000*	15.41425	.001	-115.8839	-22.1161	
		Insulin Glargin	-100.50000*	15.41425	.000	-147.3839	-53.6161	
		Glibenclamide	-198.83333*	15.41425	.000	-245.7172	-151.9494	
	Donepezil	Negative Control	474.25000*	15.41425	.000	427.3661	521.1339	
		Positive Control	-153.93333*	15.41425	.000	-200.8172	-107.0494	
		Metformin	69.00000*	15.41425	.001	22.1161	115.8839	
		Insulin Glargin	-31.50000	15.41425	.343	-78.3839	15.3839	
		Glibenclamide	-129.83333*	15.41425	.000	-176.7172	-82.9494	
	Insulin Glargin	Negative Control	505.75000*	15.41425	.000	458.8661	552.6339	
		Positive Control	-122.43333*	15.41425	.000	-169.3172	-75.5494	
		Metformin	100.50000*	15.41425	.000	53.6161	147.3839	
		Donepezil	31.50000	15.41425	.343	-15.3839	78.3839	
		Glibenclamide	-98.33333*	15.41425	.000	-145.2172	-51.4494	
	Glibenclamide	Negative Control	604.08333*	15.41425	.000	557.1994	650.9672	
		Positive Control	-24.10000	15.41425	.628	-70.9839	22.7839	
		Metformin	198.83333*	15.41425	.000	151.9494	245.7172	
		Donepezil	129.83333*	15.41425	.000	82.9494	176.7172	
		Insulin Glargin	98.33333*	15.41425	.000	51.4494	145.2172	
Serum Total Plasma Tau	Negative Control	Positive Control	-117.90000*	6.47288	.000	-137.5879	-98.2121	
		Metformin	-67.23333*	6.47288	.000	-86.9212	-47.5455	
		Donepezil	-92.13333*	6.47288	.000	-111.8212	-72.4455	
		Insulin Glargin	-70.96667*	6.47288	.000	-90.6545	-51.2788	
		Glibenclamide	-83.96667*	6.47288	.000	-103.6545	-64.2788	
	Positive Control	Negative Control	117.90000*	6.47288	.000	98.2121	137.5879	
		Metformin	50.66667*	6.47288	.000	30.9788	70.3545	
		Donepezil	25.76667*	6.47288	.005	6.0788	45.4545	
		Insulin Glargin	46.93333*	6.47288	.000	27.2455	66.6212	
		Glibenclamide	33.93333*	6.47288	.000	14.2455	53.6212	
	Metformin	Negative Control	67.23333*	6.47288	.000	47.5455	86.9212	
		Positive Control	-50.66667*	6.47288	.000	-70.3545	-30.9788	
		Donepezil	-24.90000*	6.47288	.007	-44.5879	-5.2121	
		Insulin Glargin	-3.73333	6.47288	.992	-23.4212	15.9545	
		Glibenclamide	-16.73333	6.47288	.132	-36.4212	2.9545	
	Donepezil	Negative Control	92.13333*	6.47288	.000	72.4455	111.8212	
		Positive Control	-25.76667*	6.47288	.005	-45.4545	-6.0788	
		Metformin	24.90000*	6.47288	.007	5.2121	44.5879	
		Insulin Glargin	21.16667*	6.47288	.029	1.4788	40.8545	
		Glibenclamide	8.16667	6.47288	.803	-11.5212	27.8545	
	Insulin Glargin	Negative Control	70.96667*	6.47288	.000	51.2788	90.6545	
		Positive Control	-46.93333*	6.47288	.000	-66.6212	-27.2455	
		Metformin	3.73333	6.47288	.992	-15.9545	23.4212	
		Donepezil	-21.16667*	6.47288	.029	-40.8545	-1.4788	
		Glibenclamide	-13.00000	6.47288	.361	-32.6879	6.6879	
	Glibenclamide	Negative Control	83.96667*	6.47288	.000	64.2788	103.6545	
		Positive Control	-33.93333*	6.47288	.000	-53.6212	-14.2455	
		Metformin	16.73333	6.47288	.132	-2.9545	36.4212	
		Donepezil	-8.16667	6.47288	.803	-27.8545	11.5212	
		Insulin Glargin	13.00000	6.47288	.361	-6.6879	32.6879	
Serum Neurofilament Light	Negative Control	Positive Control	-14.99667*	.71909	.000	-17.1838	-12.8095	
		Metformin	-6.41333*	.71909	.000	-8.6005	-4.2262	
		Donepezil	-4.01333*	.71909	.000	-6.2005	-1.8262	
		Insulin Glargin	.22500	.71909	1.000	-1.9622	2.4122	
		Glibenclamide	-.96333	.71909	.761	-3.1505	1.2238	
	Positive Control	Negative Control	14.99667*	.71909	.000	12.8095	17.1838	
		Metformin	8.58333*	.71909	.000	6.3962	10.7705	
		Donepezil	10.98333*	.71909	.000	8.7962	13.1705	
		Insulin Glargin	15.22167*	.71909	.000	13.0345	17.4088	
		Glibenclamide	14.03333*	.71909	.000	11.8462	16.2205	
	Metformin	Negative Control	6.41333*	.71909	.000	4.2262	8.6005	
		Positive Control	-8.58333*	.71909	.000	-10.7705	-6.3962	
		Donepezil	2.40000*	.71909	.025	.2128	4.5872	
		Insulin Glargin	6.63833*	.71909	.000	4.4512	8.8255	
		Glibenclamide	5.45000*	.71909	.000	3.2628	7.6372	
	Donepezil	Negative Control	4.01333*	.71909	.000	1.8262	6.2005	
		Positive Control	-10.98333*	.71909	.000	-13.1705	-8.7962	
		Metformin	-2.40000*	.71909	.025	-4.5872	-.2128	
		Insulin Glargin	4.23833*	.71909	.000	2.0512	6.4255	
		Glibenclamide	3.05000*	.71909	.002	.8628	5.2372	
	Insulin Glargin	Negative Control	-.22500	.71909	1.000	-2.4122	1.9622	
		Positive Control	-15.22167*	.71909	.000	-17.4088	-13.0345	
		Metformin	-6.63833*	.71909	.000	-8.8255	-4.4512	
		Donepezil	-4.23833*	.71909	.000	-6.4255	-2.0512	
		Glibenclamide	-1.18833	.71909	.572	-3.3755	.9988	
	Glibenclamide	Negative Control	.96333	.71909	.761	-1.2238	3.1505	
		Positive Control	-14.03333*	.71909	.000	-16.2205	-11.8462	
		Metformin	-5.45000*	.71909	.000	-7.6372	-3.2628	
		Donepezil	-3.05000*	.71909	.002	-5.2372	-.8628	
		Insulin Glargin	1.18833	.71909	.572	-.9988	3.3755	
Brain Amyloid Beta 1 42	Negative Control	Positive Control	-692.00000*	13.38132	.000	-732.7005	-651.2995	
		Metformin	-357.16667*	13.38132	.000	-397.8672	-316.4661	
		Donepezil	-445.76667*	13.38132	.000	-486.4672	-405.0661	
		Insulin Glargin	-460.66667*	13.38132	.000	-501.3672	-419.9661	
		Glibenclamide	-617.73333*	13.38132	.000	-658.4339	-577.0328	
	Positive Control	Negative Control	692.00000*	13.38132	.000	651.2995	732.7005	
		Metformin	334.83333*	13.38132	.000	294.1328	375.5339	
		Donepezil	246.23333*	13.38132	.000	205.5328	286.9339	
		Insulin Glargin	231.33333*	13.38132	.000	190.6328	272.0339	
		Glibenclamide	74.26667*	13.38132	.000	33.5661	114.9672	
	Metformin	Negative Control	357.16667*	13.38132	.000	316.4661	397.8672	
		Positive Control	-334.83333*	13.38132	.000	-375.5339	-294.1328	
		Donepezil	-88.60000*	13.38132	.000	-129.3005	-47.8995	
		Insulin Glargin	-103.50000*	13.38132	.000	-144.2005	-62.7995	
		Glibenclamide	-260.56667*	13.38132	.000	-301.2672	-219.8661	
	Donepezil	Negative Control	445.76667*	13.38132	.000	405.0661	486.4672	
		Positive Control	-246.23333*	13.38132	.000	-286.9339	-205.5328	
		Metformin	88.60000*	13.38132	.000	47.8995	129.3005	
		Insulin Glargin	-14.90000	13.38132	.872	-55.6005	25.8005	
		Glibenclamide	-171.96667*	13.38132	.000	-212.6672	-131.2661	
	Insulin Glargin	Negative Control	460.66667*	13.38132	.000	419.9661	501.3672	
		Positive Control	-231.33333*	13.38132	.000	-272.0339	-190.6328	
		Metformin	103.50000*	13.38132	.000	62.7995	144.2005	
		Donepezil	14.90000	13.38132	.872	-25.8005	55.6005	
		Glibenclamide	-157.06667*	13.38132	.000	-197.7672	-116.3661	
	Glibenclamide	Negative Control	617.73333*	13.38132	.000	577.0328	658.4339	
		Positive Control	-74.26667*	13.38132	.000	-114.9672	-33.5661	
		Metformin	260.56667*	13.38132	.000	219.8661	301.2672	
		Donepezil	171.96667*	13.38132	.000	131.2661	212.6672	
		Insulin Glargin	157.06667*	13.38132	.000	116.3661	197.7672	
Brain Nitric Oxide	Negative Control	Positive Control	-22.42667*	.95908	.000	-25.3438	-19.5095	
		Metformin	-2.18833	.95908	.233	-5.1055	.7288	
		Donepezil	-1.54833	.95908	.596	-4.4655	1.3688	
		Insulin Glargin	-19.22833*	.95908	.000	-22.1455	-16.3112	
		Glibenclamide	-16.96167*	.95908	.000	-19.8788	-14.0445	
	Positive Control	Negative Control	22.42667*	.95908	.000	19.5095	25.3438	
		Metformin	20.23833*	.95908	.000	17.3212	23.1555	
		Donepezil	20.87833*	.95908	.000	17.9612	23.7955	
		Insulin Glargin	3.19833*	.95908	.025	.2812	6.1155	
		Glibenclamide	5.46500*	.95908	.000	2.5479	8.3821	
	Metformin	Negative Control	2.18833	.95908	.233	-.7288	5.1055	
		Positive Control	-20.23833*	.95908	.000	-23.1555	-17.3212	
		Donepezil	.64000	.95908	.984	-2.2771	3.5571	
		Insulin Glargin	-17.04000*	.95908	.000	-19.9571	-14.1229	
		Glibenclamide	-14.77333*	.95908	.000	-17.6905	-11.8562	
	Donepezil	Negative Control	1.54833	.95908	.596	-1.3688	4.4655	
		Positive Control	-20.87833*	.95908	.000	-23.7955	-17.9612	
		Metformin	-.64000	.95908	.984	-3.5571	2.2771	
		Insulin Glargin	-17.68000*	.95908	.000	-20.5971	-14.7629	
		Glibenclamide	-15.41333*	.95908	.000	-18.3305	-12.4962	
	Insulin Glargin	Negative Control	19.22833*	.95908	.000	16.3112	22.1455	
		Positive Control	-3.19833*	.95908	.025	-6.1155	-.2812	
		Metformin	17.04000*	.95908	.000	14.1229	19.9571	
		Donepezil	17.68000*	.95908	.000	14.7629	20.5971	
		Glibenclamide	2.26667	.95908	.201	-.6505	5.1838	
	Glibenclamide	Negative Control	16.96167*	.95908	.000	14.0445	19.8788	
		Positive Control	-5.46500*	.95908	.000	-8.3821	-2.5479	
		Metformin	14.77333*	.95908	.000	11.8562	17.6905	
		Donepezil	15.41333*	.95908	.000	12.4962	18.3305	
		Insulin Glargin	-2.26667	.95908	.201	-5.1838	.6505	
Brain Acetyl choline Esterase	Negative Control	Positive Control	-43.42000*	1.25075	.000	-47.2243	-39.6157	
		Metformin	-4.76167*	1.25075	.008	-8.5660	-.9574	
		Donepezil	-12.32833*	1.25075	.000	-16.1326	-8.5240	
		Insulin Glargin	-12.14500*	1.25075	.000	-15.9493	-8.3407	
		Glibenclamide	-30.81167*	1.25075	.000	-34.6160	-27.0074	
	Positive Control	Negative Control	43.42000*	1.25075	.000	39.6157	47.2243	
		Metformin	38.65833*	1.25075	.000	34.8540	42.4626	
		Donepezil	31.09167*	1.25075	.000	27.2874	34.8960	
		Insulin Glargin	31.27500*	1.25075	.000	27.4707	35.0793	
		Glibenclamide	12.60833*	1.25075	.000	8.8040	16.4126	
	Metformin	Negative Control	4.76167*	1.25075	.008	.9574	8.5660	
		Positive Control	-38.65833*	1.25075	.000	-42.4626	-34.8540	
		Donepezil	-7.56667*	1.25075	.000	-11.3710	-3.7624	
		Insulin Glargin	-7.38333*	1.25075	.000	-11.1876	-3.5790	
		Glibenclamide	-26.05000*	1.25075	.000	-29.8543	-22.2457	
	Donepezil	Negative Control	12.32833*	1.25075	.000	8.5240	16.1326	
		Positive Control	-31.09167*	1.25075	.000	-34.8960	-27.2874	
		Metformin	7.56667*	1.25075	.000	3.7624	11.3710	
		Insulin Glargin	.18333	1.25075	1.000	-3.6210	3.9876	
		Glibenclamide	-18.48333*	1.25075	.000	-22.2876	-14.6790	
	Insulin Glargin	Negative Control	12.14500*	1.25075	.000	8.3407	15.9493	
		Positive Control	-31.27500*	1.25075	.000	-35.0793	-27.4707	
		Metformin	7.38333*	1.25075	.000	3.5790	11.1876	
		Donepezil	-.18333	1.25075	1.000	-3.9876	3.6210	
		Glibenclamide	-18.66667*	1.25075	.000	-22.4710	-14.8624	
	Glibenclamide	Negative Control	30.81167*	1.25075	.000	27.0074	34.6160	
		Positive Control	-12.60833*	1.25075	.000	-16.4126	-8.8040	
		Metformin	26.05000*	1.25075	.000	22.2457	29.8543	
		Donepezil	18.48333*	1.25075	.000	14.6790	22.2876	
		Insulin Glargin	18.66667*	1.25075	.000	14.8624	22.4710	
Brain Malondialdehyde	Negative Control	Positive Control	-3.96500*	.21716	.000	-4.6255	-3.3045	
		Metformin	-.61667	.21716	.078	-1.2772	.0438	
		Donepezil	-2.79167*	.21716	.000	-3.4522	-2.1312	
		Insulin Glargin	-2.29333*	.21716	.000	-2.9538	-1.6328	
		Glibenclamide	-3.20000*	.21716	.000	-3.8605	-2.5395	
	Positive Control	Negative Control	3.96500*	.21716	.000	3.3045	4.6255	
		Metformin	3.34833*	.21716	.000	2.6878	4.0088	
		Donepezil	1.17333*	.21716	.000	.5128	1.8338	
		Insulin Glargin	1.67167*	.21716	.000	1.0112	2.3322	
		Glibenclamide	.76500*	.21716	.016	.1045	1.4255	
	Metformin	Negative Control	.61667	.21716	.078	-.0438	1.2772	
		Positive Control	-3.34833*	.21716	.000	-4.0088	-2.6878	
		Donepezil	-2.17500*	.21716	.000	-2.8355	-1.5145	
		Insulin Glargin	-1.67667*	.21716	.000	-2.3372	-1.0162	
		Glibenclamide	-2.58333*	.21716	.000	-3.2438	-1.9228	
	Donepezil	Negative Control	2.79167*	.21716	.000	2.1312	3.4522	
		Positive Control	-1.17333*	.21716	.000	-1.8338	-.5128	
		Metformin	2.17500*	.21716	.000	1.5145	2.8355	
		Insulin Glargin	.49833	.21716	.227	-.1622	1.1588	
		Glibenclamide	-.40833	.21716	.433	-1.0688	.2522	
	Insulin Glargin	Negative Control	2.29333*	.21716	.000	1.6328	2.9538	
		Positive Control	-1.67167*	.21716	.000	-2.3322	-1.0112	
		Metformin	1.67667*	.21716	.000	1.0162	2.3372	
		Donepezil	-.49833	.21716	.227	-1.1588	.1622	
		Glibenclamide	-.90667*	.21716	.003	-1.5672	-.2462	
	Glibenclamide	Negative Control	3.20000*	.21716	.000	2.5395	3.8605	
		Positive Control	-.76500*	.21716	.016	-1.4255	-.1045	
		Metformin	2.58333*	.21716	.000	1.9228	3.2438	
		Donepezil	.40833	.21716	.433	-.2522	1.0688	
		Insulin Glargin	.90667*	.21716	.003	.2462	1.5672	
Brain Beta Secretase	Negative Control	Positive Control	-745.66667*	6.35640	.000	-765.0003	-726.3331	
		Metformin	-129.21667*	6.35640	.000	-148.5503	-109.8831	
		Donepezil	-70.68333*	6.35640	.000	-90.0169	-51.3497	
		Insulin Glargin	-201.62167*	6.35640	.000	-220.9553	-182.2881	
		Glibenclamide	-629.16667*	6.35640	.000	-648.5003	-609.8331	
	Positive Control	Negative Control	745.66667*	6.35640	.000	726.3331	765.0003	
		Metformin	616.45000*	6.35640	.000	597.1164	635.7836	
		Donepezil	674.98333*	6.35640	.000	655.6497	694.3169	
		Insulin Glargin	544.04500*	6.35640	.000	524.7114	563.3786	
		Glibenclamide	116.50000*	6.35640	.000	97.1664	135.8336	
	Metformin	Negative Control	129.21667*	6.35640	.000	109.8831	148.5503	
		Positive Control	-616.45000*	6.35640	.000	-635.7836	-597.1164	
		Donepezil	58.53333*	6.35640	.000	39.1997	77.8669	
		Insulin Glargin	-72.40500*	6.35640	.000	-91.7386	-53.0714	
		Glibenclamide	-499.95000*	6.35640	.000	-519.2836	-480.6164	
	Donepezil	Negative Control	70.68333*	6.35640	.000	51.3497	90.0169	
		Positive Control	-674.98333*	6.35640	.000	-694.3169	-655.6497	
		Metformin	-58.53333*	6.35640	.000	-77.8669	-39.1997	
		Insulin Glargin	-130.93833*	6.35640	.000	-150.2719	-111.6047	
		Glibenclamide	-558.48333*	6.35640	.000	-577.8169	-539.1497	
	Insulin Glargin	Negative Control	201.62167*	6.35640	.000	182.2881	220.9553	
		Positive Control	-544.04500*	6.35640	.000	-563.3786	-524.7114	
		Metformin	72.40500*	6.35640	.000	53.0714	91.7386	
		Donepezil	130.93833*	6.35640	.000	111.6047	150.2719	
		Glibenclamide	-427.54500*	6.35640	.000	-446.8786	-408.2114	
	Glibenclamide	Negative Control	629.16667*	6.35640	.000	609.8331	648.5003	
		Positive Control	-116.50000*	6.35640	.000	-135.8336	-97.1664	
		Metformin	499.95000*	6.35640	.000	480.6164	519.2836	
		Donepezil	558.48333*	6.35640	.000	539.1497	577.8169	
		Insulin Glargin	427.54500*	6.35640	.000	408.2114	446.8786	
Brain PMAPt	Negative Control	Positive Control	-28.17000*	1.24741	.000	-31.9641	-24.3759	
		Metformin	-7.88667*	1.24741	.000	-11.6808	-4.0926	
		Donepezil	-17.86167*	1.24741	.000	-21.6558	-14.0676	
		Insulin Glargin	-2.15333	1.24741	.526	-5.9474	1.6408	
		Glibenclamide	-4.43667*	1.24741	.015	-8.2308	-.6426	
	Positive Control	Negative Control	28.17000*	1.24741	.000	24.3759	31.9641	
		Metformin	20.28333*	1.24741	.000	16.4892	24.0774	
		Donepezil	10.30833*	1.24741	.000	6.5142	14.1024	
		Insulin Glargin	26.01667*	1.24741	.000	22.2226	29.8108	
		Glibenclamide	23.73333*	1.24741	.000	19.9392	27.5274	
	Metformin	Negative Control	7.88667*	1.24741	.000	4.0926	11.6808	
		Positive Control	-20.28333*	1.24741	.000	-24.0774	-16.4892	
		Donepezil	-9.97500*	1.24741	.000	-13.7691	-6.1809	
		Insulin Glargin	5.73333*	1.24741	.001	1.9392	9.5274	
		Glibenclamide	3.45000	1.24741	.091	-.3441	7.2441	
	Donepezil	Negative Control	17.86167*	1.24741	.000	14.0676	21.6558	
		Positive Control	-10.30833*	1.24741	.000	-14.1024	-6.5142	
		Metformin	9.97500*	1.24741	.000	6.1809	13.7691	
		Insulin Glargin	15.70833*	1.24741	.000	11.9142	19.5024	
		Glibenclamide	13.42500*	1.24741	.000	9.6309	17.2191	
	Insulin Glargin	Negative Control	2.15333	1.24741	.526	-1.6408	5.9474	
		Positive Control	-26.01667*	1.24741	.000	-29.8108	-22.2226	
		Metformin	-5.73333*	1.24741	.001	-9.5274	-1.9392	
		Donepezil	-15.70833*	1.24741	.000	-19.5024	-11.9142	
		Glibenclamide	-2.28333	1.24741	.463	-6.0774	1.5108	
	Glibenclamide	Negative Control	4.43667*	1.24741	.015	.6426	8.2308	
		Positive Control	-23.73333*	1.24741	.000	-27.5274	-19.9392	
		Metformin	-3.45000	1.24741	.091	-7.2441	.3441	
		Donepezil	-13.42500*	1.24741	.000	-17.2191	-9.6309	
		Insulin Glargin	2.28333	1.24741	.463	-1.5108	6.0774	
Day0BS	Negative Control	Positive Control	1.33333	1.70294	.968	-3.8463	6.5130	
		Metformin	-.33333	1.70294	1.000	-5.5130	4.8463	
		Donepezil	3.83333	1.70294	.245	-1.3463	9.0130	
		Insulin Glargin	-1.16667	1.70294	.982	-6.3463	4.0130	
		Glibenclamide	2.00000	1.70294	.845	-3.1796	7.1796	
	Positive Control	Negative Control	-1.33333	1.70294	.968	-6.5130	3.8463	
		Metformin	-1.66667	1.70294	.921	-6.8463	3.5130	
		Donepezil	2.50000	1.70294	.686	-2.6796	7.6796	
		Insulin Glargin	-2.50000	1.70294	.686	-7.6796	2.6796	
		Glibenclamide	.66667	1.70294	.999	-4.5130	5.8463	
	Metformin	Negative Control	.33333	1.70294	1.000	-4.8463	5.5130	
		Positive Control	1.66667	1.70294	.921	-3.5130	6.8463	
		Donepezil	4.16667	1.70294	.173	-1.0130	9.3463	
		Insulin Glargin	-.83333	1.70294	.996	-6.0130	4.3463	
		Glibenclamide	2.33333	1.70294	.744	-2.8463	7.5130	
	Donepezil	Negative Control	-3.83333	1.70294	.245	-9.0130	1.3463	
		Positive Control	-2.50000	1.70294	.686	-7.6796	2.6796	
		Metformin	-4.16667	1.70294	.173	-9.3463	1.0130	
		Insulin Glargin	-5.00000	1.70294	.063	-10.1796	.1796	
		Glibenclamide	-1.83333	1.70294	.887	-7.0130	3.3463	
	Insulin Glargin	Negative Control	1.16667	1.70294	.982	-4.0130	6.3463	
		Positive Control	2.50000	1.70294	.686	-2.6796	7.6796	
		Metformin	.83333	1.70294	.996	-4.3463	6.0130	
		Donepezil	5.00000	1.70294	.063	-.1796	10.1796	
		Glibenclamide	3.16667	1.70294	.445	-2.0130	8.3463	
	Glibenclamide	Negative Control	-2.00000	1.70294	.845	-7.1796	3.1796	
		Positive Control	-.66667	1.70294	.999	-5.8463	4.5130	
		Metformin	-2.33333	1.70294	.744	-7.5130	2.8463	
		Donepezil	1.83333	1.70294	.887	-3.3463	7.0130	
		Insulin Glargin	-3.16667	1.70294	.445	-8.3463	2.0130	
Day3BS	Negative Control	Positive Control	-4.00000	10.02451	.999	-34.4905	26.4905	
		Metformin	-4.66667	10.02451	.997	-35.1571	25.8238	
		Donepezil	-.33333	10.02451	1.000	-30.8238	30.1571	
		Insulin Glargin	-1.00000	10.02451	1.000	-31.4905	29.4905	
		Glibenclamide	-7.50000	10.02451	.974	-37.9905	22.9905	
	Positive Control	Negative Control	4.00000	10.02451	.999	-26.4905	34.4905	
		Metformin	-.66667	10.02451	1.000	-31.1571	29.8238	
		Donepezil	3.66667	10.02451	.999	-26.8238	34.1571	
		Insulin Glargin	3.00000	10.02451	1.000	-27.4905	33.4905	
		Glibenclamide	-3.50000	10.02451	.999	-33.9905	26.9905	
	Metformin	Negative Control	4.66667	10.02451	.997	-25.8238	35.1571	
		Positive Control	.66667	10.02451	1.000	-29.8238	31.1571	
		Donepezil	4.33333	10.02451	.998	-26.1571	34.8238	
		Insulin Glargin	3.66667	10.02451	.999	-26.8238	34.1571	
		Glibenclamide	-2.83333	10.02451	1.000	-33.3238	27.6571	
	Donepezil	Negative Control	.33333	10.02451	1.000	-30.1571	30.8238	
		Positive Control	-3.66667	10.02451	.999	-34.1571	26.8238	
		Metformin	-4.33333	10.02451	.998	-34.8238	26.1571	
		Insulin Glargin	-.66667	10.02451	1.000	-31.1571	29.8238	
		Glibenclamide	-7.16667	10.02451	.979	-37.6571	23.3238	
	Insulin Glargin	Negative Control	1.00000	10.02451	1.000	-29.4905	31.4905	
		Positive Control	-3.00000	10.02451	1.000	-33.4905	27.4905	
		Metformin	-3.66667	10.02451	.999	-34.1571	26.8238	
		Donepezil	.66667	10.02451	1.000	-29.8238	31.1571	
		Glibenclamide	-6.50000	10.02451	.986	-36.9905	23.9905	
	Glibenclamide	Negative Control	7.50000	10.02451	.974	-22.9905	37.9905	
		Positive Control	3.50000	10.02451	.999	-26.9905	33.9905	
		Metformin	2.83333	10.02451	1.000	-27.6571	33.3238	
		Donepezil	7.16667	10.02451	.979	-23.3238	37.6571	
		Insulin Glargin	6.50000	10.02451	.986	-23.9905	36.9905	
Day10BS	Negative Control	Positive Control	-19.50000	6.71027	.068	-39.9099	.9099	
		Metformin	-17.16667	6.71027	.139	-37.5766	3.2433	
		Donepezil	-16.00000	6.71027	.194	-36.4099	4.4099	
		Insulin Glargin	-27.83333*	6.71027	.003	-48.2433	-7.4234	
		Glibenclamide	-21.33333*	6.71027	.036	-41.7433	-.9234	
	Positive Control	Negative Control	19.50000	6.71027	.068	-.9099	39.9099	
		Metformin	2.33333	6.71027	.999	-18.0766	22.7433	
		Donepezil	3.50000	6.71027	.995	-16.9099	23.9099	
		Insulin Glargin	-8.33333	6.71027	.813	-28.7433	12.0766	
		Glibenclamide	-1.83333	6.71027	1.000	-22.2433	18.5766	
	Metformin	Negative Control	17.16667	6.71027	.139	-3.2433	37.5766	
		Positive Control	-2.33333	6.71027	.999	-22.7433	18.0766	
		Donepezil	1.16667	6.71027	1.000	-19.2433	21.5766	
		Insulin Glargin	-10.66667	6.71027	.611	-31.0766	9.7433	
		Glibenclamide	-4.16667	6.71027	.989	-24.5766	16.2433	
	Donepezil	Negative Control	16.00000	6.71027	.194	-4.4099	36.4099	
		Positive Control	-3.50000	6.71027	.995	-23.9099	16.9099	
		Metformin	-1.16667	6.71027	1.000	-21.5766	19.2433	
		Insulin Glargin	-11.83333	6.71027	.503	-32.2433	8.5766	
		Glibenclamide	-5.33333	6.71027	.966	-25.7433	15.0766	
	Insulin Glargin	Negative Control	27.83333*	6.71027	.003	7.4234	48.2433	
		Positive Control	8.33333	6.71027	.813	-12.0766	28.7433	
		Metformin	10.66667	6.71027	.611	-9.7433	31.0766	
		Donepezil	11.83333	6.71027	.503	-8.5766	32.2433	
		Glibenclamide	6.50000	6.71027	.924	-13.9099	26.9099	
	Glibenclamide	Negative Control	21.33333*	6.71027	.036	.9234	41.7433	
		Positive Control	1.83333	6.71027	1.000	-18.5766	22.2433	
		Metformin	4.16667	6.71027	.989	-16.2433	24.5766	
		Donepezil	5.33333	6.71027	.966	-15.0766	25.7433	
		Insulin Glargin	-6.50000	6.71027	.924	-26.9099	13.9099	
Day30BS	Negative Control	Positive Control	-.66667	14.88704	1.000	-45.9470	44.6137	
		Metformin	-12.66667	14.88704	.955	-57.9470	32.6137	
		Donepezil	-9.66667	14.88704	.986	-54.9470	35.6137	
		Insulin Glargin	-36.83333	14.88704	.164	-82.1137	8.4470	
		Glibenclamide	-22.66667	14.88704	.653	-67.9470	22.6137	
	Positive Control	Negative Control	.66667	14.88704	1.000	-44.6137	45.9470	
		Metformin	-12.00000	14.88704	.964	-57.2803	33.2803	
		Donepezil	-9.00000	14.88704	.990	-54.2803	36.2803	
		Insulin Glargin	-36.16667	14.88704	.178	-81.4470	9.1137	
		Glibenclamide	-22.00000	14.88704	.680	-67.2803	23.2803	
	Metformin	Negative Control	12.66667	14.88704	.955	-32.6137	57.9470	
		Positive Control	12.00000	14.88704	.964	-33.2803	57.2803	
		Donepezil	3.00000	14.88704	1.000	-42.2803	48.2803	
		Insulin Glargin	-24.16667	14.88704	.590	-69.4470	21.1137	
		Glibenclamide	-10.00000	14.88704	.984	-55.2803	35.2803	
	Donepezil	Negative Control	9.66667	14.88704	.986	-35.6137	54.9470	
		Positive Control	9.00000	14.88704	.990	-36.2803	54.2803	
		Metformin	-3.00000	14.88704	1.000	-48.2803	42.2803	
		Insulin Glargin	-27.16667	14.88704	.466	-72.4470	18.1137	
		Glibenclamide	-13.00000	14.88704	.950	-58.2803	32.2803	
	Insulin Glargin	Negative Control	36.83333	14.88704	.164	-8.4470	82.1137	
		Positive Control	36.16667	14.88704	.178	-9.1137	81.4470	
		Metformin	24.16667	14.88704	.590	-21.1137	69.4470	
		Donepezil	27.16667	14.88704	.466	-18.1137	72.4470	
		Glibenclamide	14.16667	14.88704	.929	-31.1137	59.4470	
	Glibenclamide	Negative Control	22.66667	14.88704	.653	-22.6137	67.9470	
		Positive Control	22.00000	14.88704	.680	-23.2803	67.2803	
		Metformin	10.00000	14.88704	.984	-35.2803	55.2803	
		Donepezil	13.00000	14.88704	.950	-32.2803	58.2803	
		Insulin Glargin	-14.16667	14.88704	.929	-59.4470	31.1137	
Day60BS	Negative Control	Positive Control	-72.83333*	21.29424	.020	-137.6018	-8.0649	
		Metformin	-2.50000	21.29424	1.000	-67.2684	62.2684	
		Donepezil	-36.50000	21.29424	.534	-101.2684	28.2684	
		Insulin Glargin	-57.83333	21.29424	.101	-122.6018	6.9351	
		Glibenclamide	-30.00000	21.29424	.722	-94.7684	34.7684	
	Positive Control	Negative Control	72.83333*	21.29424	.020	8.0649	137.6018	
		Metformin	70.33333*	21.29424	.027	5.5649	135.1018	
		Donepezil	36.33333	21.29424	.538	-28.4351	101.1018	
		Insulin Glargin	15.00000	21.29424	.980	-49.7684	79.7684	
		Glibenclamide	42.83333	21.29424	.360	-21.9351	107.6018	
	Metformin	Negative Control	2.50000	21.29424	1.000	-62.2684	67.2684	
		Positive Control	-70.33333*	21.29424	.027	-135.1018	-5.5649	
		Donepezil	-34.00000	21.29424	.607	-98.7684	30.7684	
		Insulin Glargin	-55.33333	21.29424	.129	-120.1018	9.4351	
		Glibenclamide	-27.50000	21.29424	.787	-92.2684	37.2684	
	Donepezil	Negative Control	36.50000	21.29424	.534	-28.2684	101.2684	
		Positive Control	-36.33333	21.29424	.538	-101.1018	28.4351	
		Metformin	34.00000	21.29424	.607	-30.7684	98.7684	
		Insulin Glargin	-21.33333	21.29424	.914	-86.1018	43.4351	
		Glibenclamide	6.50000	21.29424	1.000	-58.2684	71.2684	
	Insulin Glargin	Negative Control	57.83333	21.29424	.101	-6.9351	122.6018	
		Positive Control	-15.00000	21.29424	.980	-79.7684	49.7684	
		Metformin	55.33333	21.29424	.129	-9.4351	120.1018	
		Donepezil	21.33333	21.29424	.914	-43.4351	86.1018	
		Glibenclamide	27.83333	21.29424	.779	-36.9351	92.6018	
	Glibenclamide	Negative Control	30.00000	21.29424	.722	-34.7684	94.7684	
		Positive Control	-42.83333	21.29424	.360	-107.6018	21.9351	
		Metformin	27.50000	21.29424	.787	-37.2684	92.2684	
		Donepezil	-6.50000	21.29424	1.000	-71.2684	58.2684	
		Insulin Glargin	-27.83333	21.29424	.779	-92.6018	36.9351	

*. The mean difference is significant at the 0.05 level.	


Homogeneous Subsets


Serum Glucose	
Tukey HSDa  	
Group	N	Subset for alpha = 0.05	
		1	2	3	4	
Negative Control	6	132.1667				
Metformin	6		326.8333			
Glibenclamide	6			450.1667		
Donepezil	6				676.3333	
Insulin Glargin	6				695.6667	
Positive Control	6				761.0000	
Sig.		1.000	1.000	1.000	.073	

Means for groups in homogeneous subsets are displayed.	
a. Uses Harmonic Mean Sample Size = 6.000.	


Serum HBA1c	
Tukey HSDa  	
Group	N	Subset for alpha = 0.05	
		1	2	
Negative Control	6	5.40		
Glibenclamide	6		8.53	
Metformin	6		8.57	
Insulin Glargin	6		9.40	
Positive Control	6		9.68	
Donepezil	6		10.13	
Sig.		1.000	.238	

Means for groups in homogeneous subsets are displayed.	
a. Uses Harmonic Mean Sample Size = 6.000.	


Serum Total Cholesterol	
Tukey HSDa  	
Group	N	Subset for alpha = 0.05	
		1	2	3	
Negative Control	6	41.1667			
Glibenclamide	6	41.1667			
Donepezil	6		56.8333		
Metformin	6		60.0000		
Insulin Glargin	6		61.1667		
Positive Control	6			76.5000	
Sig.		1.000	.903	1.000	

Means for groups in homogeneous subsets are displayed.	
a. Uses Harmonic Mean Sample Size = 6.000.	


Serum Triglyceride	
Tukey HSDa  	
Group	N	Subset for alpha = 0.05	
		1	2	
Negative Control	6	60.1667		
Metformin	6	61.0000		
Donepezil	6	65.3333		
Insulin Glargin	6	68.1667		
Glibenclamide	6	74.1667		
Positive Control	6		128.3333	
Sig.		.959	1.000	

Means for groups in homogeneous subsets are displayed.	
a. Uses Harmonic Mean Sample Size = 6.000.	


Serum Low Density Lipid	
Tukey HSDa  	
Group	N	Subset for alpha = 0.05	
		1	
Glibenclamide	6	5.7333	
Negative Control	6	10.7667	
Donepezil	6	11.6333	
Metformin	6	13.3333	
Insulin Glargin	6	13.5333	
Positive Control	6	14.3667	
Sig.		.358	

Means for groups in homogeneous subsets are displayed.	
a. Uses Harmonic Mean Sample Size = 6.000.	


Serum High Density Lipid	
Tukey HSDa  	
Group	N	Subset for alpha = 0.05	
		1	2	
Glibenclamide	6	24.5000		
Negative Control	6	29.5000	29.5000	
Positive Control	6		34.1667	
Donepezil	6		34.1667	
Metformin	6		34.8333	
Insulin Glargin	6		36.0000	
Sig.		.374	.134	

Means for groups in homogeneous subsets are displayed.	
a. Uses Harmonic Mean Sample Size = 6.000.	


Serum Interlukin 6	
Tukey HSDa  	
Group	N	Subset for alpha = 0.05	
		1	2	3	
Negative Control	6	32.2833			
Positive Control	6		81.6000		
Metformin	6		92.1000	92.1000	
Glibenclamide	6		95.9833	95.9833	
Donepezil	6		106.9167	106.9167	
Insulin Glargin	6			117.4000	
Sig.		1.000	.082	.082	

Means for groups in homogeneous subsets are displayed.	
a. Uses Harmonic Mean Sample Size = 6.000.	


Serum Tumor Necrosis Factor	
Tukey HSDa  	
Group	N	Subset for alpha = 0.05	
		1	2	3	
Negative Control	6	11.9333			
Donepezil	6	21.2000			
Metformin	6	22.2667			
Glibenclamide	6	28.3833	28.3833		
Positive Control	6		44.7167	44.7167	
Insulin Glargin	6			63.0667	
Sig.		.123	.128	.065	

Means for groups in homogeneous subsets are displayed.	
a. Uses Harmonic Mean Sample Size = 6.000.	


Serom Amyloid Beta 1 42	
Tukey HSDa  	
Group	N	Subset for alpha = 0.05	
		1	2	3	4	
Negative Control	6	71.9167				
Metformin	6		477.1667			
Donepezil	6			546.1667		
Insulin Glargin	6			577.6667		
Glibenclamide	6				676.0000	
Positive Control	6				700.1000	
Sig.		1.000	1.000	.343	.628	

Means for groups in homogeneous subsets are displayed.	
a. Uses Harmonic Mean Sample Size = 6.000.	


Serum Total Plasma Tau	
Tukey HSDa  	
Group	N	Subset for alpha = 0.05	
		1	2	3	4	
Negative Control	6	22.9333				
Metformin	6		90.1667			
Insulin Glargin	6		93.9000			
Glibenclamide	6		106.9000	106.9000		
Donepezil	6			115.0667		
Positive Control	6				140.8333	
Sig.		1.000	.132	.803	1.000	

Means for groups in homogeneous subsets are displayed.	
a. Uses Harmonic Mean Sample Size = 6.000.	


Serum Neurofilament Light	
Tukey HSDa  	
Group	N	Subset for alpha = 0.05	
		1	2	3	4	
Insulin Glargin	6	3.1950				
Negative Control	6	3.4200				
Glibenclamide	6	4.3833				
Donepezil	6		7.4333			
Metformin	6			9.8333		
Positive Control	6				18.4167	
Sig.		.572	1.000	1.000	1.000	

Means for groups in homogeneous subsets are displayed.	
a. Uses Harmonic Mean Sample Size = 6.000.	


Brain Amyloid Beta 1 42	
Tukey HSDa  	
Group	N	Subset for alpha = 0.05	
		1	2	3	4	5	
Negative Control	6	86.0000					
Metformin	6		443.1667				
Donepezil	6			531.7667			
Insulin Glargin	6			546.6667			
Glibenclamide	6				703.7333		
Positive Control	6					778.0000	
Sig.		1.000	1.000	.872	1.000	1.000	

Means for groups in homogeneous subsets are displayed.	
a. Uses Harmonic Mean Sample Size = 6.000.	


Brain Nitric Oxide	
Tukey HSDa  	
Group	N	Subset for alpha = 0.05	
		1	2	3	
Negative Control	6	7.4383			
Donepezil	6	8.9867			
Metformin	6	9.6267			
Glibenclamide	6		24.4000		
Insulin Glargin	6		26.6667		
Positive Control	6			29.8650	
Sig.		.233	.201	1.000	

Means for groups in homogeneous subsets are displayed.	
a. Uses Harmonic Mean Sample Size = 6.000.	


Brain Acetyl choline Esterase	
Tukey HSDa  	
Group	N	Subset for alpha = 0.05	
		1	2	3	4	5	
Negative Control	6	41.4883					
Metformin	6		46.2500				
Insulin Glargin	6			53.6333			
Donepezil	6			53.8167			
Glibenclamide	6				72.3000		
Positive Control	6					84.9083	
Sig.		1.000	1.000	1.000	1.000	1.000	

Means for groups in homogeneous subsets are displayed.	
a. Uses Harmonic Mean Sample Size = 6.000.	


Brain Malondialdehyde	
Tukey HSDa  	
Group	N	Subset for alpha = 0.05	
		1	2	3	4	
Negative Control	6	.3583				
Metformin	6	.9750				
Insulin Glargin	6		2.6517			
Donepezil	6		3.1500	3.1500		
Glibenclamide	6			3.5583		
Positive Control	6				4.3233	
Sig.		.078	.227	.433	1.000	

Means for groups in homogeneous subsets are displayed.	
a. Uses Harmonic Mean Sample Size = 6.000.	


Brain Beta Secretase	
Tukey HSDa  	
Group	N	Subset for alpha = 0.05	
		1	2	3	4	5	6	
Negative Control	6	116.3667						
Donepezil	6		187.0500					
Metformin	6			245.5833				
Insulin Glargin	6				317.9883			
Glibenclamide	6					745.5333		
Positive Control	6						862.0333	
Sig.		1.000	1.000	1.000	1.000	1.000	1.000	

Means for groups in homogeneous subsets are displayed.	
a. Uses Harmonic Mean Sample Size = 6.000.	


Brain PMAPt	
Tukey HSDa  	
Group	N	Subset for alpha = 0.05	
		1	2	3	4	5	
Negative Control	6	16.3800					
Insulin Glargin	6	18.5333	18.5333				
Glibenclamide	6		20.8167	20.8167			
Metformin	6			24.2667			
Donepezil	6				34.2417		
Positive Control	6					44.5500	
Sig.		.526	.463	.091	1.000	1.000	

Means for groups in homogeneous subsets are displayed.	
a. Uses Harmonic Mean Sample Size = 6.000.	


Day0BS	
Tukey HSDa  	
Group	N	Subset for alpha = 0.05	
		1	
Donepezil	6	76.5000	
Glibenclamide	6	78.3333	
Positive Control	6	79.0000	
Negative Control	6	80.3333	
Metformin	6	80.6667	
Insulin Glargin	6	81.5000	
Sig.		.063	

Means for groups in homogeneous subsets are displayed.	
a. Uses Harmonic Mean Sample Size = 6.000.	


Day3BS	
Tukey HSDa  	
Group	N	Subset for alpha = 0.05	
		1	
Negative Control	6	175.0000	
Donepezil	6	175.3333	
Insulin Glargin	6	176.0000	
Positive Control	6	179.0000	
Metformin	6	179.6667	
Glibenclamide	6	182.5000	
Sig.		.974	

Means for groups in homogeneous subsets are displayed.	
a. Uses Harmonic Mean Sample Size = 6.000.	


Day10BS	
Tukey HSDa  	
Group	N	Subset for alpha = 0.05	
		1	2	
Negative Control	6	217.5000		
Donepezil	6	233.5000	233.5000	
Metformin	6	234.6667	234.6667	
Positive Control	6	237.0000	237.0000	
Glibenclamide	6		238.8333	
Insulin Glargin	6		245.3333	
Sig.		.068	.503	

Means for groups in homogeneous subsets are displayed.	
a. Uses Harmonic Mean Sample Size = 6.000.	


Day30BS	
Tukey HSDa  	
Group	N	Subset for alpha = 0.05	
		1	
Negative Control	6	353.3333	
Positive Control	6	354.0000	
Donepezil	6	363.0000	
Metformin	6	366.0000	
Glibenclamide	6	376.0000	
Insulin Glargin	6	390.1667	
Sig.		.164	

Means for groups in homogeneous subsets are displayed.	
a. Uses Harmonic Mean Sample Size = 6.000.	


Day60BS	
Tukey HSDa  	
Group	N	Subset for alpha = 0.05	
		1	2	
Negative Control	6	503.3333		
Metformin	6	505.8333		
Glibenclamide	6	533.3333	533.3333	
Donepezil	6	539.8333	539.8333	
Insulin Glargin	6	561.1667	561.1667	
Positive Control	6		576.1667	
Sig.		.101	.360	

Means for groups in homogeneous subsets are displayed.	
a. Uses Harmonic Mean Sample Size = 6.000.	
