# Supplementary material for: Effects of antidiabetic agents on Alzheimer’s disease biomarkers in experimentally induced hyperglycemic rat model by streptozocin
Source: PLoS One. 2022 Jul 8;17(7):e0271138. doi: 10.1371/journal.pone.0271138 (PMC9269384; doi:10.1371/journal.pone.0271138)
Supplement: Attachment — Submitted filename: Response to the reviewer docx copy.docx [file pone.0271138.s002.docx]

Dear editor,

# I am re-submitting the revised version of the manuscript "Effects of Antidiabetic Agents on Alzheimer’s Disease Biomarkers in Experimentally Induced Hyperglycemic Rat Model by Streptozocin", which is authored by Shatw Kh. Ali and Rojgar H. Ali for consideration for publication in Plos One Journal.

# As per reviewers comments we edited the texts that were unclear and revised the English language to enhance the comprehension and the flow.

For your information, along with this cover letter, the following files have been submitted:

1. Revised manuscript showing changes through review tracker in MS word.
2. A clean version of the revised manuscripts (no review tracker).

Additionally, in this letter you will find our one-by-one responses (highlighted in yellow) to the reviewer’s questions and comments.

Kind Regards,

Shatw Kh. Ali

Reviewer #1:

In this study, authors compared the effectiveness of multiple antidiabetic drugs in a model of experimental diabetes mellitus induced by intraperitoneal (ip) injection of streptozocin (STZ). A myriad of serum markers were then assessed in controls and type 2 diabetes animals.

1) The first issue with the study is that authors state in the Abstract that ip STZ injection induces Alzheimer's disease model. This is not true, because the sporadic AD-type model is induced by intracerebroventricular STZ injection. Authors should specify that with ip injection of STZ they induced diabetes-like changes and not AD.

We do agree with you, the paragraph was changed accordingly. Please see line 24-27.

Also, the model has been changed to Hyperglycemic Rat Model.

2) Why do authors use abbreviations in brackets multiple times in the text? And why should "vascular dementia" and "cerebrovascular disease" be abbreviated if it is used only once in the whole manuscript?

The whole manuscript was revised for such mistakes. The changes can be seen in the revised version with the review tracker.

3) Method: it is insufficient to state the weight of the animals. What was the age of animals? What conditions were they kept in (temperature, lighting, humidity)? How many rats per cage? Was there an acclimatization period? All of this information is missing.

We have added all the missing information to the method section. The changes can be seen in the revised version with the review tracker. Please see line 94-102

Authors state that blood glucose was measured "in the eyes" (line 110). I suppose this means that it was a retro-orbital blood collection? Was anaesthesia used for this? Procedure description is incomplete.

As you recommended, this paragraph was changed and the procedure is completely described. Please see line (126-135)

What do authors mean in lines 110-111 by "and Accu Check Glucometer and left for 2 months". So animals were injected ip with STZ and left for 2 months, and only after that treated with various compounds for 21 consecutive days? It is unclear. A picture with experimental design would greatly aid to understanding the timeline of the procedures done by authors.

Accu Check Glucometer is an instant blood glucose monitoring machine used to measure the blood glucose of the rats during that period. The animals were left for 60 days till all the diabetes complications were apparent. These paragraphs were all revised and described in detail to aid to understand the timeline of the procedure better and we think there is no need for a picture after the revision. Please see line (128-139)

4) Results: abbreviation HSD is not defined. The use of the same symbol to depict differences between different groups seems misleading.

Tukey's Honest Significant Difference (HSD) test is a post hoc test commonly used to assess the significance of differences between pairs of group means. While we do agree with you and we have removed it from the paragraphs.

Consider using * for comparison with negative control and # for comparison with positive control.

We have changed the comparison using * for negative control and # for positive control upon your request.

Line 136 states that mean +/- S.E.M. was used, but in line 171 authors mention S.D. Which was it then, SEM or SD?

That mistake was fixed and both are SEM and not SD. Please see line (199-200)

In lines 178-179 authors state that HDL levels significantly differed in glibenclamide-treated STZ rats compared to all treatment groups. This is not shown in Table 1.

That mistake was fixed and shown with a # in **table 2** in the revised manuscript.

Why would authors report ANOVA results for LDL levels but not for other markers? ANOVA tells us if the values between groups differ, and then by using a post-hoc test we show the values of which specific groups are significantly different. ANOVA is not really reported in this manuscript.

ANOVA results have been added to table 2 and table 3 for all the markers upon your request.

5) Discussion is too large. Description of each marker (and the rationale for assessing their levels) should be stated in the Introduction!

Discussion was condensed as much as possible upon your request but the description and the level of the markers were stated in the discussion.

In lines 261-264 authors describe the changes seen after peripheral injection of STZ, while citing an article on the effects of centrally injected STZ (Singh et al., 2015).

This paragraph was revised and we have changed the reference as well. Please see line (264-266)

Overall, manuscript looks like it was not read thoroughly before submitting, as indicated by discrepancies in the use of abbreviation and grammar.

The manuscript was revised to enhance the English language. The changes can be seen in the revised version with the review tracker.

Moreover, authors state that they induced AD, which they did not.

We agree with you, which is why the model has been updated to the Hyperglycemic Rat Model, which we believe it is more accurate.

Reviewer #2:

In “Effects of Antidiabetic Agents on Alzheimer’s Disease Biomarkers in Rats with Experimentally Induced Diabetes: Randomized Experimental Design”, the authors present a study utilizing a hyperglycemic rat model that also exhibits elevations in several biomarkers for Alzheimer’s Disease (AD). After 3 weeks of administering various diabetes and AD medications to different groups of rats, several metabolic and cognitive parameters are quantified in the serum and brain extracts. The findings of the study are relevant and potentially useful to the scientific and medical community. However, many concerning issues with the study first need to be addressed.

The authors perform intraperitoneal (i.p.) injections of STZ in 5 to 6 month old male rats to generate models of hyperglycemia and AD. While this procedure is perfectly valid for generating hyperglycemia, the legitimacy of this method for generating AD is questionable. The references the authors cite for inducing AD by this procedure do not support their methodology. For example, in reference 12, Zhang et al. induced learning and memory impairments by injecting STZ (i.p.) into neonatal mice. In order to induce such effects in adult mice, Zhang et al. administered intracerebroventricular STZ injections. Furthermore, although the current authors do show several biomarkers associated with AD (e.g. amyloid beta) to be upregulated in their experimental rat model, no cognitive or behavioral tests are ever performed. I suggest that the authors present this model as a hyperglycemic model rather than a model of AD, unless data can be added to the manuscript that verifies learning and memory deficits.

We agree with you, we have updated the model to the Hyperglycemic Rat Model, which we believe it is more accurate. That paragraph was revised and we have changed the reference as well. Please see line (264-266)

After induction of the rat model, the authors divided the rodents into various treatment groups and administered different therapeutic agents to each group for 21 days. Most of the utilized agents were anti-diabetic agents with one exception – donepezil – a typical AD agent. Two control groups were included (no STZ and STZ without further treatment). The authors then sacrificed the mice and quantified the amounts of various biomarkers in the serum and brain extracts using ELISAs. While the data presented are potentially useful to the scientific and medical community, several issues were identified that should be addressed.

• First, it appears as though the authors did not measure blood glucose in all of the rats before starting the various treatments. While unlikely, it is possible that the STZ did not effectively induce hyperglycemia in all rodents, and this could potentially skew the apparent results of any treatment that those rats received. If the authors did indeed verify hyperglycemia in all of the rats before beginning treatment, those data should be provided in the manuscript.

Table 1 has been added to the manuscript which shows the blood glucose level before and after the injection of streptozocin over several days of the experiment.

• The results from the ELISAs are all presented in two tables, which are well organized and clear. However, the description of the results in the text is overly repetitive. Much of this text could be condensed. Also, I am not sure if comparing each treatment to every other treatment for each biomarker in the statistical analysis adds value to the study. It is somewhat confusing. In my opinion, simply comparing the STZ control to the no STZ control and then each treatment group to the STZ control would be sufficient. That is how the data are presented in the tables.

We agree with you, much of the text has been condensed in the result section. Comparison is only done between negative control group and the positive control group and then between positive control group and the treatment groups after the revision.

Lastly, the authors continuously refer to the model as an AD model, but it is not clear that the model or the diabetes treatments would be relevant to AD in the context of normoglycemia. As mentioned above, it would be best to present this model as a model of hyperglycemia with upregulation of several AD associated biomarkers. The findings may be most relevant to diabetes and diabetes linked AD.

We have updated the model to the Hyperglycemic Rat Model, which we believe it is more accurate.

In addition to the scientific or presentation concerns mentioned above, the authors also need to heavily revise the manuscript for language and grammatical errors.

The manuscript was revised to enhance the English language. The changes can be seen in the revised version with the review tracker.

There are too many errors for this reviewer to correct at this stage. However, a few key points are mentioned below:

• Many words are inappropriately capitalized (e.g. Glucose, Hemoglobin, Total Cholesterol, etc…).

These mistakes have been fixed in the whole manuscript.

• Once the abbreviation for a word is given in parenthesis, the parenthesis does not need to be used throughout the manuscript. For example, the authors routinely refer to AD as (AD).

These mistakes have been fixed in the whole manuscript.

• Lines 64 and 65: The statement “(AD) and vascular dementia have both been demonstrated to enhance the risk of cognitive impairment and dementia in people with type 2 (DM) (8,9).” is incorrect. After reviewing the sources cited (8 and 9), I suspect the authors meant “T2DM has been shown to enhance the risk of cognitive impairment, AD, and vascular dementia”.

These mistakes have been fixed in the whole manuscript.

• Line 114: Typo: “oral daily doses of Insulin”. The authors later state that insulin was given subcutaneously, which is the correct way to administer insulin glargine.

This mistake has been fixed.

• Line 263: STZ does not induce insulin resistance.

This mistake has been fixed.
Despite the many concerns raised in the above review, I do think that the findings have scientific merit and can be useful to the scientific community if presented in the right context.

Many thanks for your helpful comments, almost all the mistakes are fixed and almost all the manuscript has been revised upon your request. We hope that now the manuscript is in the right context.
