# Supplementary material for: Effects of antidiabetic agents on Alzheimer’s disease biomarkers in experimentally induced hyperglycemic rat model by streptozocin
Source: PLoS One. 2022 Jul 8;17(7):e0271138. doi: 10.1371/journal.pone.0271138 (PMC9269384; doi:10.1371/journal.pone.0271138)
Supplement: Attachment — Submitted filename: Response to the reviewer docx 1.docx [file pone.0271138.s003.docx]

Dear editor,

# I am re-submitting the revised version of the manuscript "Effects of Antidiabetic Agents on Alzheimer’s Disease Biomarkers in Experimentally Induced Hyperglycemic Rat Model by Streptozocin", which is authored by Shatw Kh. Ali and Rojgar H. Ali for consideration for publication in Plos One Journal.

# As per reviewers comments we edited the texts that were unclear.

For your information, along with this cover letter, the following files have been submitted:

1. Revised manuscript showing changes through review tracker in MS word.
2. A clean version of the revised manuscripts (no review tracker).

Additionally, in this letter you will find our one-by-one responses (highlighted in yellow) to the reviewer’s questions and comments.

Kind Regards,

Shatw Kh. Ali

Reviewer #1: (No Response)

Reviewer #2: In the latest revision of the manuscript entitled “Effects of Antidiabetic Agents on Alzheimer’s Disease Biomarkers in Experimentally Induced Hyperglycemic Rat Model by Streptozocin” the authors did address many of the concerns raised in the original review. In particular, the authors no longer refer to the model as an AD model, but rather as a model of DM, which was the major issue raised by both reviewers. The authors also added information about the blood glucose levels of the rats prior to treatment induction, as requested by Reviewer 2. They also attempted to simplify and clarify some of the results section by mostly comparing the treatment groups to the positive control group, as suggested. The English is also very much improved in the revised version. While the manuscript has indeed improved, it is my opinion that further improvements can be made. I have added further suggestions, line by line, below.

Line 5: “Randomized Experimental Design” likely not needed in the title.

We have removed it from the title.

Line 21: “… resulting in insulin resistance”. Insulin resistance is only present in T2DM, so this should be removed from the general statement about diabetes. The next line differentiates between T1DM and T2DM.

We have removed that line.

Line 63: “.. resulting in insulin resistance.” Not all diabetes has insulin resistance – just T2DM.

We have removed that line from the abstract and the introduction as well.

Line 71: Replace “diabetes” with “DM”.

Diabetes is replaced by DM in line 69 in the latest revised manuscript.

Line 77: Did you mean to use the word “hypertensive”, or did you mean “hyperglycemic”?

This mistake has been fixed and the right word is hyperglycemic not hypertensive. Please see line 75 in the revised manuscript.

Line 105: Change to “rat IL6 enzyme-linked assay (ELISA) kits” instead of “….. rat kits..”

We have changed that line, please see line 103 in the revised manuscript.

Line 106: The parentheses around the company name is not needed.

Parentheses have been removed around the company name in line 103-105 in the revised manuscript.

Line 121: Consider rephrasing to make it clear that only groups II, III, IV, V, and VI received the STZ. Then the last sentence of this paragraph (Line 124 – 125) could be deleted.

Line 118-119 is revised in the revised manuscript, and the rest is removed upon your request.

Line 138: This sentence is confusing. Do you mean that according to previous studies, all of the complications should be apparent within 4 – 8 weeks? Or do you mean that all of the diabetes complications in this study were apparent in 4 – 8 weeks?

Line 135-137 has been rephrased, we meant ‘according to previous studies...’

Table 1: Consider using averages and standard deviations rather than ranges.

The blood glucose ranges are removed and replaced by mean blood glucose level ± standard deviations in (Table 1) in the revised manuscript.

Line 144: Consider replacing “were treated” with “began treatment” since the treatment was continuous.

Were treated is replaced by began treatment in the revised manuscript in line 142.

Lines 149 – 151: The grammar needs correction. Suggestion: “The oral medications were dissolved in normal saline, and the correct dosage was calculated. The medications were administered to the animals through oral gavage, except for insulin, which was injected subcutaneously.”

Line 147-149 is corrected upon your request in the revised manuscript.

Line 154: How were the serum tubes stored? At what temperature and for how long?

(Serum were kept in tubes and were kept in the freezer at -23°C for five days). This line has been added in the revised manuscript in line 152-153.

Line 158: “… homogenized by hand.” Was a mortar and pestle used? Or a tissue grinder? Can you specify? Also consider using the term “brain extracts” instead of “fluid of brain tissues”.

Mortar and pestle were used for the brain homogenization, this has been added in the revised manuscript in line 157. Also, ‘‘fluid of brain tissue’’ is replaced by brain extracts.

Line 160: Consider replacing the phrase “for a few days” with something more formal like “three days” or “approximately 3 days”.

Few days is replaced by the exact number of days which was five days in line 159.

Line 176: Consider changing “serum blood level” to “serum biomarkers”. Also, the colon is not needed in the section titles.

Serum blood level is replaced by serum biomarkers and the colon is removed from the title section in line 175.

Lines 180 – 182: The way this is phrased is confusing. Are you saying that all forms of treatment significantly reduced blood glucose levels compared to the positive control, except for donepezil and insulin glargine?

This line is fixed and rephrased upon your request in line 179-181.

Line 183: There is no need to keep repeating “(pre-treated with STZ)”, as that should be clear already when you refer to the positive control.

That has been removed in repeated places.

Lines 188 – 190: Can you specify what kind of difference (e.g. reduction)?

That is right, the difference was a reduction, the line 188-190 is rephrased.

A similar issue exists in lines 191 – 195.

Line 194-195 is also rephrased and we have specified the type of difference.

Lines 196 – 206: Since there are no differences in LDL or HDL, can these paragraphs be combined/condensed?

These two paragraphs are condensed upon your request in line 196-203 but there is difference in HDL level and fixed in the latest revised manuscript.

Line 204 – 205: If the ANOVA showed no significant difference, the analysis should stop there. How can one continue with the post hoc?

The post hoc test is performed only for HDL since there is a difference in the ANOVA result, but removed for LDL because there is no difference. Please see line 196-202.

Line 207 – 211: Same issue as above with the post hoc following a non-significant ANOVA. Please clarify.

Regarding IL6, the ANOVA result indicates a significant difference P_=_ 0.0001; thus, a post hoc test is conducted. Insulin glargine raised IL6 levels significantly higher than the positive control group, as shown. Please see line 203-207

Line 214: The wording is confusing. I can see in the table that the insulin group had higher TNFa than the positive control, but this is not clear in the text.

The paragraph is rephrased please see line 208-212.

Line 223: Again, the wording is confusing. Are you saying that all treatment groups exhibited significantly lower levels of tau in the serum compared to the positive control?

This line is rephrased please see line 221-224.

Line 226: Similar issue as that raised for line 223.

That line is also rephrased, please see line 217-220.

Line 236 and 237: What does it mean when you say “the difference between all the groups was significant”? Are you saying that all of the groups were significantly different than the positive control? That’s how the data are presented in the table. Also, can you specify in the text whether they were higher or lower? This type of issue occurs several times in the results section. Please clarify throughout the manuscript.

All the results have been revised and we have specified the change in the revised manuscript upon your request.

Table 3: The column titles are not consistent with those in Table 2. In Table 2, the column titles do not contain “STZ + ..” for the treatment groups. This is a minor issue.

This has been fixed and STZ is added for all the treatment groups.

Line 261: The wording here makes it sound like STZ is naturally occurring in mammals, which is not the case.

The wording has been changed in this line upon your request. Please see line 275 in the revised manuscript.

Line 272: Consider rephrasing. As it currently reads, it suggests that hyperglycemia is a cause of increased glucose levels, and those are the same things.

This line has been changed, please see line 284-285.

Line 285 – 287: Consider revising. As mentioned earlier, if the ANOVA was not significant, no further post hoc should be performed.

As mentioned earlier, there is difference in HDL level while not in LDL, so post hoc test is only done for HDL level and removed for LDL upon your request. Please see line 298-301

Lines 304 – 307: This is not clear. Consider rephrasing.

This line is fixed and rephrased. Please see line 303-305

Line 312: The text says “all the treatment groups” and only later mentions that glibenclamide was an exception. Consider rephrasing.

This line is rephrased, please see line 315-318.

General Comments:

The structure of the article and the way the results are presented is somewhat confusing or hard to follow. It is not clear why these particular medications were chosen or why these exact biomarkers were chosen. It is hard to keep track of what affects what as you are reading through the manuscript. The tables can be a useful reference for anyone interested in how particular medications have been shown to affect particular biomarkers, so I think the results can be useful.

Because DM and Alzheimer's disease are both time-related, many diabetic patients also have Alzheimer's. It's important to compare the effects of various anti-diabetic medicines on Alzheimer's disease biomarkers.

A good place to bring things together and discuss the overarching relevance of the results would be the discussion section. However, I feel this is currently lacking, as the discussion section largely just restates the results.

The discussion has also been revised, and additional information have been provided in the revised manuscript.

Furthermore, while I believe that the statistics are sound for the most part, there are several instances in the results section where the data are presented in a way that does not fit with the described statistical methods. These instances are mentioned above in the “line by line” section.

All the comments are revised very well upon your request in the revised manuscript.

Many thanks for your helpful comments, almost all the mistakes are fixed and almost all the manuscript has been revised upon your request. We hope that now the manuscript is in the right context.
